# The Destructive Tree Pathogen *Phytophthora ramorum* Originates from the Laurosilva Forests of East Asia

**DOI:** 10.3390/jof7030226

**Published:** 2021-03-18

**Authors:** Thomas Jung, Marília Horta Jung, Joan F. Webber, Koji Kageyama, Ayaka Hieno, Hayato Masuya, Seiji Uematsu, Ana Pérez-Sierra, Anna R. Harris, Jack Forster, Helen Rees, Bruno Scanu, Sneha Patra, Tomáš Kudláček, Josef Janoušek, Tamara Corcobado, Ivan Milenković, Zoltán Nagy, Ildikó Csorba, József Bakonyi, Clive M. Brasier

**Affiliations:** 1 Phytophthora Research Centre, Faculty of Forestry and Wood Technology, Mendel University in Brno, 613 00 Brno, Czech Republic; marilia.jung@mendelu.cz (M.H.J.); snehampatra@gmail.com (S.P.); tomas.kudlacek@mendelu.cz (T.K.); josef.janousek@mendelu.cz (J.J.); tamara.sanchez@mendelu.cz (T.C.); ivan.milenkovic@mendelu.cz (I.M.); zoltan.nagy@mendelu.cz (Z.N.); 2 Phytophthora Research and Consultancy, 83131 Nußdorf, Germany; 3 Forest Research, Alice Holt Lodge, Farnham GU10 4LH, Surrey, UK; joan.webber@forestresearch.gov.uk (J.F.W.); ana.perez-sierra@forestresearch.gov.uk (A.P.-S.); anna.harris@forestresearch.gov.uk (A.R.H.); jack.forster@forestresearch.gov.uk (J.F.); helen.rees@bristol.ac.uk (H.R.); 4 River Basin Research Center, Gifu University, Gifu 501-1193, Japan; kageyama@green.gifu-u.ac.jp (K.K.); ayakatwb@gmail.com (A.H.); 5 Forestry and Forest Products Research Institute (FFPRI), Tsukuba, Ibaraki 305-8687, Japan; massw@ffpri.affrc.go.jp; 6 Departament of Bioregulation and Biointeraction, Laboratory of Molecular and Cellular Biology, Tokyo University of Agriculture and Technology, Fuchu, Tokyo 183-8509, Japan; fv9088@go.tuat.ac.jp; 7 Department of Agricultural Sciences, University of Sassari, 07100 Sassari, Italy; bscanu@uniss.it; 8 Laboratory of Ecological Plant Physiology, CzechGlobe, Global Change Research Institute of the Czech Academy of Sciences, 603 00 Brno, Czech Republic; 9 Centre for Agricultural Research, Plant Protection Institute, ELKH, H-1022 Budapest, Hungary; csorba.ildiko@atk.hu (I.C.); bakonyi.jozsef@atk.hu (J.B.)

**Keywords:** epidemic, lineages, phylogeny, evolutionary history, mating types, biosecurity

## Abstract

As global plant trade expands, tree disease epidemics caused by pathogen introductions are increasing. Since ca 2000, the introduced oomycete *Phytophthora ramorum* has caused devastating epidemics in Europe and North America, spreading as four ancient clonal lineages, each of a single mating type, suggesting different geographical origins. We surveyed laurosilva forests for *P. ramorum* around Fansipan mountain on the Vietnam-China border and on Shikoku and Kyushu islands, southwest Japan. The surveys yielded 71 *P. ramorum* isolates which we assigned to eight new lineages, IC1 to IC5 from Vietnam and NP1 to NP3 from Japan, based on differences in colony characteristics, gene x environment responses and multigene phylogeny. Molecular phylogenetic trees and networks revealed the eight Asian lineages were dispersed across the topology of the introduced European and North American lineages. The deepest node within *P. ramorum*, the divergence of lineages NP1 and NP2, was estimated at 0.5 to 1.6 Myr. The Asian lineages were each of a single mating type, and at some locations, lineages of “opposite” mating type were present, suggesting opportunities for inter-lineage recombination. Based on the high level of phenotypic and phylogenetic diversity in the sample populations, the coalescence results and the absence of overt host symptoms, we conclude that *P. ramorum* comprises many anciently divergent lineages native to the laurosilva forests between eastern Indochina and Japan.

## 1. Introduction

Outbreaks of destructive tree diseases caused by introduced pathogens are increasing due to the growing international trade in plants [1,2,3,4]. The origins of the causal agents are often obscure, probably because they cause little harm in their natural habitats, only becoming seriously damaging when introduced into a biogeographic zone containing similar host genera with limited genetic resistance and an environment that favours pathogen establishment and spread. In the new environments, the behaviour and population structure of introduced pathogens are often biological artefacts shaped by founder effects and episodic selection on the over-susceptible hosts [5,6]. A deeper understanding of their natural behaviour including their breeding systems, potential for adaptation and buffering by natural antagonists should come from studying them in their areas of origin. The resistance mechanisms of endemic hosts may also offer valuable insights for resistance breeding. However, when a forest pathogen causes little observable damage in its native habitats, identifying its geographic origin can be logistically challenging.

Many recently introduced tree pathogens are species of *Phytophthora* (Oomycota), a genus of around 150 described species, with possibly another 400–500 undescribed species awaiting discovery in under-researched ecosystems [7]. Since the late 1960s, forest epidemics caused by introduced Phytophthoras have increased exponentially [8]. One of the most recently damaging is *P. ramorum,* an aerial pathogen with a wide host range in its introduced state. Since ca 1990, it has caused the deaths of millions of native oaks and tanoaks in western North America (“sudden oak death”) and later of plantation-grown larch (“sudden larch death”) in the UK and other European countries [8,9,10,11,12,13]. In its introduced form, *P. ramorum* is known to occur as four anciently divergent clonal lineages, of which NA1 and NA2 were initially detected in North America and EU1 and EU2 in Europe [14,15,16,17,18]. Only the A1 mating type has been found in the EU1 and EU2 lineages (an exception in EU1 is probably due to somatic recombination [19]), and NA1 and NA2 are exclusively of A2 type. In culture, the lineages exhibit differences in colony patterns, temperature-growth responses and pathogenicity similar to those usually seen between *Phytophthora* species [20,21,22]. They are therefore assumed to have originated from different humid forest areas with Fagaceae, such as Yunnan, south west China, and other Asian regions [23], although surveys in Yunnan and in Nepal and Taiwan failed to find *P. ramorum* [24,25,26,27]. However, the discovery that *P. ramorum*’s closest phylogenetic relative, *Phytophthora lateralis*, currently epidemic on native *Chamaecyparis lawsoniana* in western USA [28], was native to *Chamaecyparis obtusa* cloud forests in Taiwan [24,29] again highlighted east Asia as a likely origin for *P. ramorum*.

Between 2016 and 2018, we conducted two expeditions to northern Vietnam, close to Yunnan, [30] and two to Shikoku and Kyushu in southwest Japan, to sample Phytophthoras in the evergreen “laurosilva” forests. Although molecular methods will detect *Phytophthora* species from various substrates, the “gold standard” is to obtain live cultures for further study. By isolating directly from lesions on naturally fallen leaves and baiting leaves, we successfully isolated *P. ramorum* from several river catchments in both Vietnam and Japan. We assessed the diversity of the isolates and their relationship to the four known lineages via detailed phenotypic and phylogenetic analyses. Our findings demonstrate that *P. ramorum* comprises numerous ancient, differently adapted lineages that are probably endemic to the laurosilva forests extending from Indochina (eastern Myanmar, northern Laos and Vietnam, and southwest China) to Japan.

## 2. Materials and Methods

### 2.1. Sampling Areas, Isolation and Identification of Phytophthora Species

**Figure 1:**
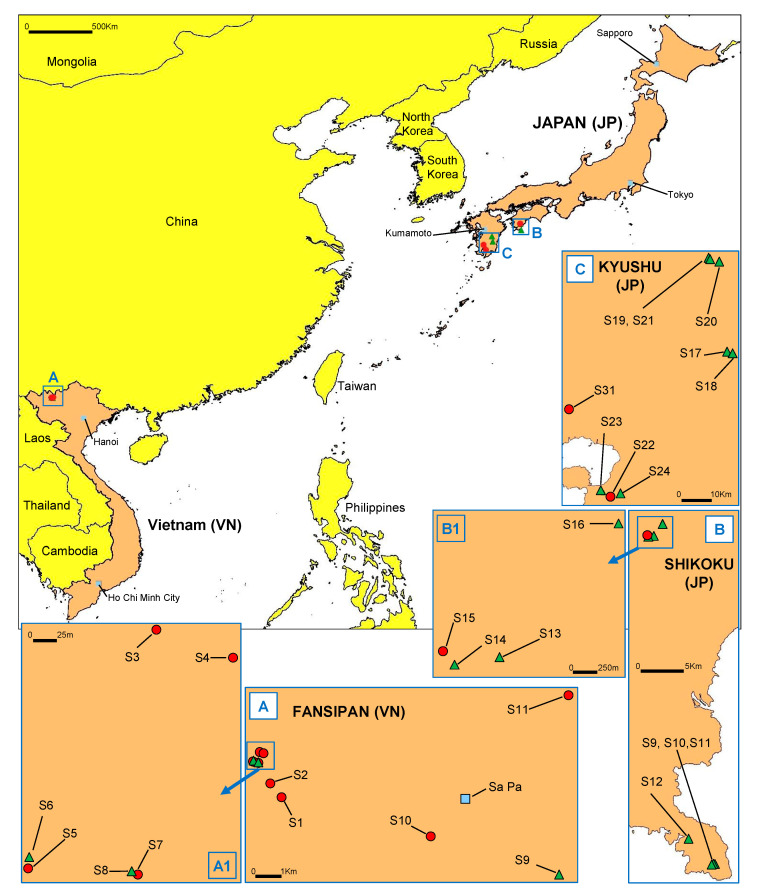
Location of areas and stream catchments (S) sampled in Vietnam and Japan. (**A**) Fansipan (VN-S1 to VN-S10) and Sau-Chua (VN-S11) areas in northern Vietnam close to the Yunnan border. (**B**) Shikoku, Japan, Tosashimizu (JP-S9 to JP-S12) and Shimanto (JP-S13 to JP-S16) areas. (**C**) Kyushu, Japan, Aya (JP-S17, JP-S18), Shiba (JP-S19 to JP-S21), Tarumizu (JP-S22 to JP-S24) and Kirishima (JP-S31) areas. Red circles indicate sample sites with *Phytophthora ramorum*; green triangles indicate sites where *P. ramorum* was not detected.

**Figure 2:**
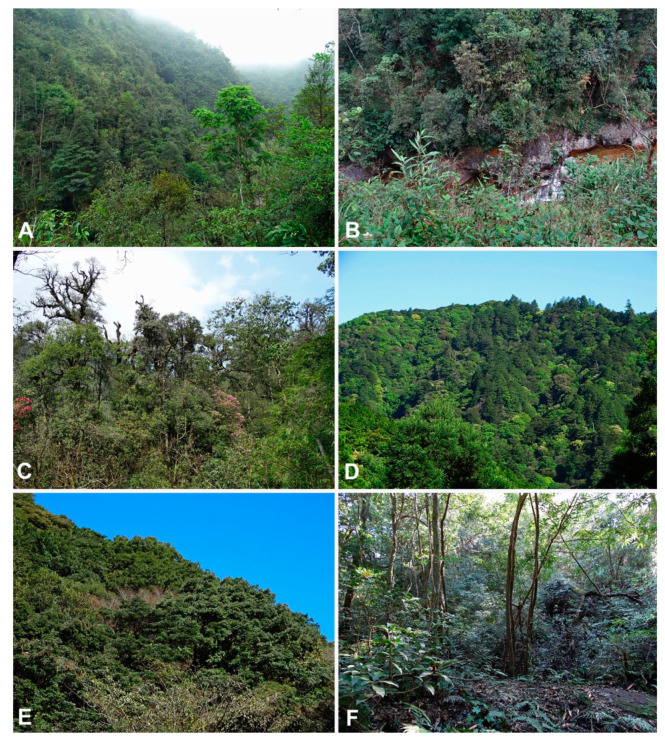
Representative laurosilva forest stands in Vietnam and Japan from which *Phytophthora ramorum* has been isolated. (**A**–**C**) *Castanopsis*-*Quercus*-*Neolitsea* dominated forests at the Fansipan in Hoàng Liên National Park, northern Vietnam. (**A**,**B**) Cat Cat River catchment (VN-S10) C. Catchment VN-S7. (**D**) *Abies*-*Chamaecyparis*-*Tsuga*-*Quercus* forest in catchment JP-S15, Shimanto area, Shikoku, Japan. (**E**,**F**) *Castanopsis*-*Lithocarpus* dominated forest in catchment JP-R22, Tarumizu area, Kyushu, Japan.

Eleven stream and river catchments were surveyed for Phytophthoras in 2016 and 2017 on the north Vietnam—Yunnan, China border within diverse laurosilva forests, ten around Fansipan Mountain in Hoàng Liên National Park and one at Sau Chua Mountain (Figures 1 and 2A–C). In addition, 17 stream and river catchments were sampled in 2017 and 2018 in diverse laurosilva forests on Shikoku and Kyushu islands, southwest Japan (Figures 1 and 2D–F).

*Phytophthora* isolations were attempted from naturally fallen necrotic leaves and flowers collected from forest streams and the forest floor around forest streams, from bait leaves floated in small rafts for 3–5 days at the surface of the forest streams, and from soil samples taken from the rhizosphere of forest trees using a standardised leaf baiting procedure. Sections of necrotic leaf tissues were plated onto selective PARPNH-agar and emerging *Phytophthora* hyphae were transferred under a stereomicroscope to V8-juice agar (V8A) [27,30,31]. *Phytophthora* isolates were identified by comparing their morphological structures on V8A at ×400 with descriptions [32,33] and by sequence analysis of the internal transcribed spacer (ITS1-5.8S-ITS2) region of the ribosomal DNA [30,34]. Isolates were stored as 5 mm diam plugs cut from a 3-weeks old V8A culture placed in 2 mL cryogenic tubes filled with sterile distilled water and kept at 8 °C in darkness.

### 2.2. Growth Media and Culture Maintenance

Carrot agar (CA) was prepared from the filtrate of 200g blended and compressed washed carrots and 15g Oxoid^®^ n°3 agar (Oxoid, Basingstoke, UK) per litre distilled water [35]. Carrot agarose medium (CAG) [36] was prepared as for CA but with 15g agarose (Invitrogen^TM^ UltraPure Agarose, Life Technologies, Inchinnan, UK). V8 juice agar (V8A) was prepared from 200 mL proprietary vegetable juice, 3g of Calcium Carbonate, 15g Oxoid^®^ n°3 agar and 800 mL distilled water. V8 2% agar (V82A) was prepared as above but with 20g Oxoid^®^ n°3 agar. Selective PARPNH agar was prepared as described by Jung et al. [37] by adding the antibiotics (10 μg/mL pimaricin, 200 μg/mL ampicillin, 10 μg/mL rifampicin, 25 μg/mL pentachloronitrobenzene (PCNB), 50 μg/mL nystatin (all from Sigma Group a.s., Lutín, Czech Republic) and 50 μg/mL hymexazol (Sankyo, Tokyo, Japan)), dissolved in 2–3 mL of 75% ethanol, to the autoclaved V8A when it had cooled to 45 °C. Agar media were autoclaved at 121 °C for 15 min and dispensed at ca 15 mL per 90 mm plate except for CAG which was hand poured at ca 10–11 mL per plate.

### 2.3. Phenotypic Studies

#### 2.3.1. Known *Phytophthora ramorum* Lineage Isolates

Three isolates of each of the four known *P. ramorum* lineages (details in [21] and Table S2) were used as lineage standards in growth rate tests, gene x environment tests, mating tests and phylogenetic analyses. These were EU1 A1 mating type isolates P1376, P1367 and P1578; EU2 A1 isolates P2460–P2462; NA1 A2 isolates P1403, P1421 and P1498; and NA2 A2 isolates P2056–P2058.

#### 2.3.2. Growth Rates and Colony Characteristics

Isolates were grown on CA plates for four days at 20 °C, close to the optimum for all the *P. ramorum* lineages [21]; then, 5-mm plugs cut from the edges of these actively growing colonies were transferred to two fresh CA plates and incubated in the dark at 20 °C. Two colony diameters were measured on each plate at right angles after two days and again after a further five days; then, the mean daily radial growth rate was calculated. Colony types were assessed after another seven days in darkness at 20 °C [35]. In addition to known lineage controls (listed above), the tests involved 56 Vietnamese and seven Japanese isolates: Vietnam IC1, 50 isolates including all isolates used in the gene x environment (g x e) stress tests and mating tests (see below); IC2, VN150, VN169, VN314; IC3, VN88; IC4, VN851; IC5, VN863; Japan NP1, JP236, JP716, JP916; NP2, JP387, JP462; NP3, JP975.

#### 2.3.3. Gene x Environment Stress Tests

V82A represents a lower level of free water availability than the 1.5% agar normally used for *Phytophthora* growth tests [21]. Plates were inoculated with 5-mm diameter plugs cut from actively growing colonies and incubated in darkness at 28.5 °C (Tests 1 and 2) or 28 °C (Test 3), close to the maximum temperature tolerated by *P. ramorum* in vitro [21]. Colony diameters less the plug diameter were measured after 12 days and mean daily radial growth rates calculated.

In addition to known lineage controls (listed above) the 21 isolates used in Test 1 were Vietnam IC1, VN57, VN75, VN120, VN122, VN160, VN163, VN216, VN283 and VN313. The 21 isolates used in Test 2 were Vietnam IC1, VN160, VN856; IC2, VN142, VN150; IC3, VN88; IC4, VN851; IC5, VN863; Japan NP1, JP236, JP716, JP1202; NP2, JP387, JP462; and NP3, JP975. The 30 isolates used in Test 3 were Vietnam IC1, VN160, VN166, VN856, VN864, VN878; IC2, VN142, VN150, VN169, VN314; IC3, VN88; IC4, VN851; IC5, VN863; Japan NP1, JP236, JP716, JP916; NP2, JP387, JP462; and NP3, JP975.

#### 2.3.4. Mating Tests

Multiple mating type tests were carried out on thinly poured CAG plates using the *P. ramorum* juvenile mycelium mixing method [38] involving serial subculturing of the isolates on CAG followed by intimate mixing of small 2–3-day old colony pieces of the “unknown” isolate with an A1 or A2 tester isolate. For 47 Vietnamese isolates, two known A1 and A2 lineage isolates were used as testers. For the seven Japanese isolates, one known A1 and A2 lineage isolate and two newly confirmed Vietnamese A1 and A2 isolates (VN160 and VN314) were used as testers. Plates were incubated for 2 weeks in darkness at 20 °C and examined for gametangia (antheridia and oogonia) through the bottom of the Petri dishes at ×100 magnification.

Resulting gametangia in fertile matings were too scarce to be examined efficiently from microscope slide preparations. Gametangial measurements and observations of oospore condition were therefore made directly through the bottom of the Petri dishes at ×100 magnification whereas photos of individual gametangia were taken later from slides at ×320 magnification. Dimensions of 20 gametangia and oospores were measured from each of the following four fertile Vietnamese A1 × A2 matings: VN122 × VN134, VN160 × VN169, VN160 × VN314 and VN57 × VN314. Fertile Vietnamese and Japanese A1 × A2 matings from which oospore viability/abortion was examined were VN122 × VN314, VN123 × VN314, VN71 × VN314, VN118 × VN314, VN281 × VN314, VN160 × VN314, VN57 × VN314, VN169 × VN160, VN57 × VN88, VN160 × VN88 and JP1202 × JP462. Oospores with a well-developed wall, a vacuole and an oily granular matrix were considered normal whereas oogonia with either no oospore, a thin-walled oospore with no contents or an oospore with glassy refractive or “scrambled” contents were considered aborted (Figure 6).

#### 2.3.5. Statistical Analyses

Differences in growth rates at 20 and 28 °C within and between the *P. ramorum* lineages were analysed in R 3.6.1 [39]. The overall effect was tested by the generalized linear model with the Gamma family and a subsequent likelihood-ratio test. Post hoc multiple comparison tests were done using the Tukey’s method in the R package emmeans [40]. All tests were performed at significance level α = 0.05.

### 2.4. DNA Isolation, Amplification and Sequencing

Mycelial DNA of 16 Vietnamese isolates from phenotype groups IC1 to IC5, seven Japanese isolates from phenotype groups NP1 to NP3, three isolates each of the four known lineages of *P. ramorum* and, as outgroup, one isolate of *P. lateralis*, the closest relative of *P. ramorum* in Clade 8c (Table S2) were extracted from pure cultures grown in pea-broth medium [41] for 7–10 d at 25 °C without shaking. Mycelium was harvested by filtration through filter paper, washed with sterile deionized water, then freeze-dried and ground to a fine powder in liquid nitrogen [33]. Total DNA was extracted using the E.Z.N.A.^®^ Fungal DNA Mini Kit (OMEGA Bio-tek, Norcross, GA) following the manufacturer’s instructions and checked for quality and quantity by spectrophotometry (NanoDrop 1000™, Thermo Fisher Scientific Inc., Waltham, MA). DNA was stored at −20 °C until further use. Seven nuclear DNA regions, the ITS, two putative effectors (*Avh120* and *Avh121*), beta-tubulin (*btub*), heat shock protein 90 (*hsp90*), a gene coding for a hypothetical protein with a glycosyl transferase group 1 domain (gwEuk.30.30.1), the indole-3-glycerol-phosphate synthase N-5′-phosphoribosyl anthranilate isomerase gene (*trp1*) and segments of five mitochondrial loci, the cytochrome c oxidase subunits 1 (*cox1*) and 2 (*cox2*) with the spacer between them, NADH dehydrogenase subunit 1 (*nadh1*), Prv8 and Prv9, were amplified and sequenced according to [15,16,34,42,43,44,45,46] using the primers and annealing temperatures given in Table S3. All PCR mixtures contained 30ng (ITS) or 100ng (all other loci) DNA, 0.2 mM dNTPs, various concentrations of Mg^2+^ and primers, and 1 U *Taq* DNA polymerase (1.5 U for *cox1* amplified with primers FM80RC/FM85) in 50 μL PCR buffer (Table S3). Cycling conditions included an initial denaturation at 94 °C for 3 min (*hsp90* and ITS) or 2.5 min (all other loci); cycles of 30 sec at 94 °C (35 times for *Avh120*, *Avh121*, gwEuk.30.30.1, *hsp90*, ITS, Prv9 and *trp1* and 40 times for *btub*, *cox1*, *cox2*, *nadh1* and Prv8), 30 sec annealing at the primer specific temperatures and elongation at 72 °C for 1 min, followed by a final extension at 72 °C for 5 min (*hsp90*) or 10 min (all other loci).

PCR products were purified and sequenced by GATC Biotech (Konstanz, Germany) in both directions with the primers used for PCR amplification (Table S3). Electropherograms were quality checked, and forward and reverse reads were compiled using Pregap4 version 1.5 and Gap version 4.10 of the Staden software package [47] or Geneious v. 11.1.2 (Biomatters Ltd., Auckland, New Zealand). Heterozygous sites observed were labelled according to the IUPAC coding system. All sequences derived in this study were deposited in GenBank and accession numbers are given in Table S2. For *Phytophthora hibernalis* from Clade 8c, the 12 gene regions were taken from whole genome shotgun sequence contigs of GenBank assembly accession GCA_012656075.1 (Table S2).

### 2.5. Phylogenetic and Coalescence Analyses

#### 2.5.1. Maximum-Likelihood, Bayesian Inference and Coalescence Analyses

Sequences of each locus were aligned using the online MAFFT v.7 (//mafft.cbrc.jp/alignment/server/ (accessed on 15 March 2021)) [48] by the G-INS-i option. Phylogenetic analyses within both the Maximum-Likelihood (ML) and Bayesian Inference (BI) framework were performed for the twelve individual gene regions and for the concatenated seven-locus nuclear (*Avh120*, *Avh121*, *btub*, gwEuk.30.30.1, *hsp90*, ITS and *trp1*) and five-locus mitochondrial (*cox1*, *cox2*, *nadh1*, Prv8 and Prv9) datasets.

Best-fit partitioning schemes for the multilocus datasets and evolutionary models (for individual loci and for each subset in the case of multilocus datasets) were selected with PartitionFinder 2 [49] using the corrected Akaike Information Criterion (AICc). Analyses were performed for both linked and unlinked branch lengths (the resulting scheme and models were exactly the same for all datasets). The program was allowed to use the largest set of models possible, comprising 84 models in total (option *models = allx;)*. All possible partitioning schemes were tested (option *search = all;*). The partitioning schemes selected by PartitionFinder 2 were used for both ML and BI analyses. Evolutionary models selected by this program were used only for the ML analysis.

ML phylogenies were inferred using the IQ-TREE software [50] and 2000 standard nonparametric bootstrap replicates were run. These were summarised via the 50% majority-rule consensus tree with the SumTrees script version 4.4.0 within the Python library DendroPy 4.4.0 [51]. Edge lengths of the target tree were computed as mean lengths for the corresponding edges in the input set of trees.

BI analyses were performed within the BEAST 2 software package [52]. All analyses were carried out using the Standard template. Automatic model selection was conducted via model averaging implemented in the bModelTest package [53]. For both concatenated datasets, relative divergence times were calculated using the uncorrelated lognormal relaxed molecular clock model [54]. Two independent runs were executed with the concatenated datasets each with the chain length 50 million generations. Every 5000th trace and tree state were sampled, and the resulting set of trace and tree samples combined using LogCombiner 2.6.1 (part of BEAST 2) with burn-in set to 25%. Parameter estimates were summarized with TreeAnnotator v.2.6.0 (part of BEAST 2) and mapped onto the 50% majority-rule consensus tree created by SumTrees 4.4.0 [51]. SumTrees was set to treat all the trees as rooted (option *force-rooted*). Node ages of the target tree were adjusted to reflect the mean ages of the respective nodes of the input trees. Relative divergence times of the *P. ramorum* lineages were calculated as a percentage of the time to the most recent common ancestor (TMRCA) of *P. ramorum* and *P. lateralis*. For comparability with the concatenated ML trees, the branch lengths of the consensus time trees were converted to substitutions per site by multiplying the length of each branch with its mean substitution rate. Conversion and tree manipulations were done in R 3.6.1 [39] utilizing functions from the packages ape [55], treeio [56] and ggtree [57,58,59]. Posterior estimates of the parameters were summarised with Tracer [60] and estimate quality assessed based on the ESS value and visual analysis of the trace plots. The minimum ESS value for adequate sampling of the parameter was 200. Both analyses resulted in the majority of the parameters including likelihood having ESS >> 1000. All trees were visualized using the R package ggtree [57,58,59] and Inkscape version 0.92.4 [61]. All datasets and trees deriving from BI and ML analyses are available from TreeBASE (ID 27745; www.treebase.org/ (accessed on 15 March 2021)).

Following Goss et al. [15] absolute lineage divergence times were estimated based on silent substitutions across the seven nuclear loci and published average nuclear silent substitution rates for angiosperms, fungi, mammals and birds of 2−7 × 10^−9^ substitutions per site per year [62,63,64,65,66,67]. In accordance with Galtier et al. [68], we refrained from using the silent mtDNA substitutions and published mtDNA substitution rates for calculating divergence times.

#### 2.5.2. Phylogenetic Network Analyses

Since phylogenetic networks are often better suited than bifurcating phylogenetic trees to correctly resolve character conflicts in population genealogies resulting from reticulation events, recombination events or homoplasy [69,70,71,72], the concatenated nuclear and mitochondrial datasets were analysed using median-joining networks (MJNs [69]) and split decomposition networks (SDNs [73]). MJNs were created with Network version 10.1.0.0 [74]. A weight of 20 was assigned to characters in which insertions or deletions had occurred and 10 for all other characters. For both concatenated datasets two MJNs were built with tolerance parameter ε = 0 and alternatively with ε = 10 to reveal less parsimonious pathways. SDNs were constructed using SplitsTree version 4.16.2 [75]. Weighted Hamming distances [76] were used with the same character weights setting as for the MJNs. Ambiguous characters were treated with the option “*match*”. The distances were normalized. The equal angle algorithm with convex hull [77] was applied. The visual appearance of all the networks was post-processed in Inkscape version 0.92.4 [61]. For rooting the networks, we followed coalescence theory in population genetics suggesting that the node with the highest number of connections and/or the most central position should be the root [70,72,78].

## 3. Results

### 3.1. Discovery of P. ramorum in East Asian Ecosystems

We obtained 505 *Phytophthora* cultures from the 11 stream and river catchments sampled on the northern Vietnam–Yunnan border within diverse Fagaceae, Ericaceae and Lauraceae forest communities at 1200–2100 m above sea level (asl), 64 of which from seven Fansipan streams and the stream at Sau Chua Mountain belonged to a sexually self-sterile *Phytophthora* later identified as *P. ramorum* based on morphological characteristics and ITS rDNA sequences (Table S1; Figure 1 and Figure 2A–C).

The 17 stream and river catchments sampled on Shikoku and Kyushu islands yielded 597 *Phytophthora* cultures. Seven isolates (1.2%), three from one catchment in the Shimanto area on Shikoku (situated in *Chamaecyparis, Castanopsis, Lithocarpus* and *Quercus* forest at 520 m asl) and four from two catchments in Kyushu’s Kirishima and Tarumizu areas (situated mainly in *Neolitsea* and *Quercus* forest at 335–530 m asl) (Figure 1 and Figure 2D–F), were also identified as *P. ramorum* (Table S1). All isolates were obtained directly from naturally fallen leaves in forest streams (or rarely the adjacent forest floor) and from leaf baits deployed in the streams (Table S1). In addition, two close relatives of *P. ramorum* from Clade 8c, *Phytophthora foliorum* and *P. lateralis*, were isolated from three sites (Table S1).

### 3.2. Phenotype Groups Among Vietnamese and Japanese P. ramorum Isolates

#### 3.2.1. Growth Rates and Colony Characteristics

**Figure 3:**
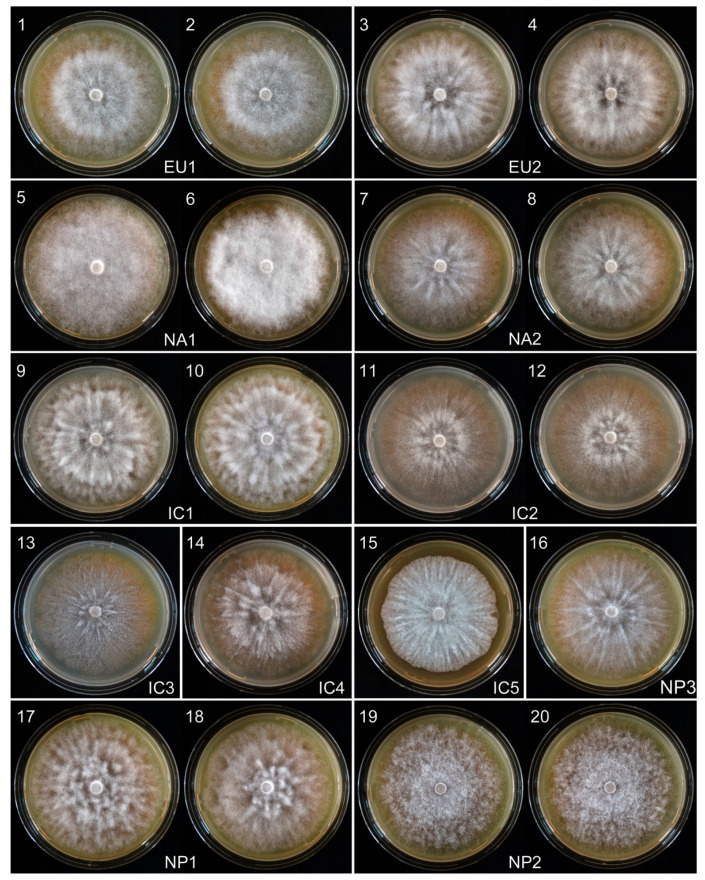
Colony morphologies of four known lineages (EU1, EU2, NA1 and NA2), five new Indochina lineages (IC1-IC5) and three new Japanese lineages (NP1-NP3) of *Phytophthora ramorum* after 7 days growth at 20 °C on CA. Isolate codes as follows: 1, P1376; 2, P1578; 3, P2460; 4, P2461; 5, P1403; 6, P1496; 7, P2056; 8, P2058; 9, VN160; 10, VN856; 11, VN150; 12, VN169; 13, VN88; 14, VN851; 15, VN863; 16, JP975; 17, JP236; 18, JP718; 19, JP387 and 20, JP462.

**Figure 4:**
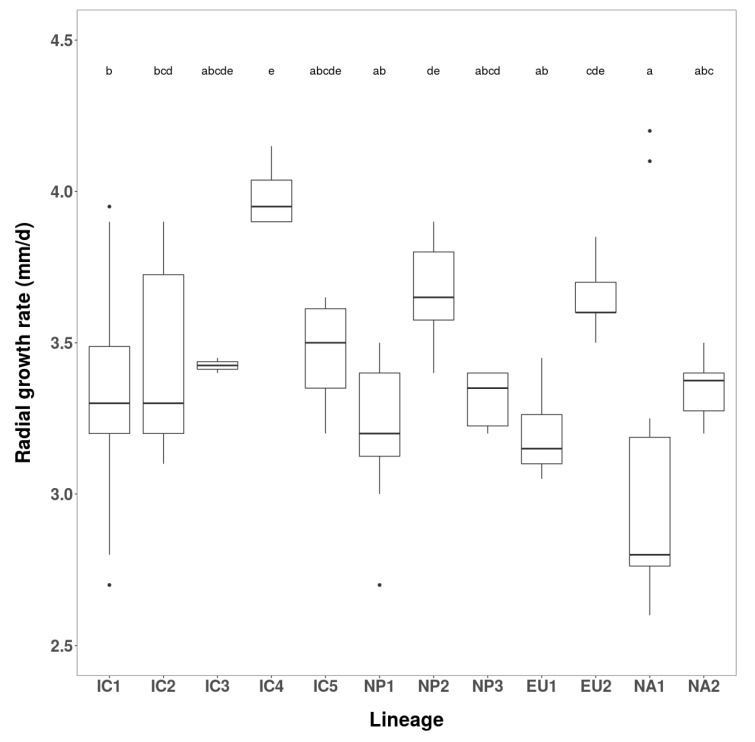
Box and whiskers diagram showing daily radial growth rates of the eight Asian phenotype groups and four known lineages of *Phytophthora ramorum* at 20 °C on carrot agar (CA). Different letters indicate statistical differences at significance level α = 0.05.

Fifty-eight Vietnamese isolates, seven Japanese isolates, and three isolates of each of the four known *P. ramorum* lineages (EU1, EU2, NA1 and NA2) were evaluated for radial growth rate and colony morphology on carrot agar (CA) at 20 °C. As previously described, the colony patterns of the known lineages were distinctive (Figure 3), and their mean growth rates were in the order EU2 > NA2 > EU1 > NA1 (Figure 4).

Vietnamese isolates exhibited five colony patterns distinct from those of the known lineages. Fifty-two isolates (91.2 %) had a common pattern, including four isolates considered minor variants. These were designated the Indochina 1 (IC1) phenotype group (Figure 3). Despite a shared colony phenotype, the IC1 isolates varied significantly in growth rate, ranging from 2.7–3.9 mm/d (Figure 4 and Figure S1) indicating multiple genotypes within the group. Mean growth rate of IC1 was similar to that of the NA2 lineage (Figure 4). Four other colony types, designated IC2, IC3, IC4 and IC5 phenotypes, were represented by just six isolates (Figure 3). IC2 comprised three isolates (VN150, VN169 and VN314) ranging in growth rate from 3.2–3.8 mm/d (Figure 4). Isolate VN314 was significantly faster growing than VN150 and VN169 (Figure S2), again indicating different genotypes. In further screening a fourth isolate, VN142, was also assigned to the IC2 group (Table 1 and Table S1). Isolates VN88 (IC3), VN851 (IC4) and VN863 (IC5) each had a unique colony pattern (Figure 3) and were faster growing than IC1 and IC2. IC4 isolate VN851, at 4.0 mm/d, was by far the fastest growing of all Vietnamese isolates, and significantly exceeded the growth rate of most other groups and lineages (Figure 4).

Japanese isolates fell into three distinct colony groups, Nippon 1 (NP1) comprising three isolates, NP2 comprising two isolates, and NP3 represented by one isolate (Figure 3). At 3.2, 3.7 and 3.5 mm/d, the mean growth rates of NP1, NP2 and NP3 were similar to those of EU1, EU2 and NA2, respectively (Figure 4). The growth rate of NP2 differed significantly from NP1 and NP3. This and their strikingly different colony patterns suggested another three lineages among the Japanese isolates.

The order of growth rates for the IC and NP groups and the known lineages was IC4 > NP2 ≈ EU2 > IC5 ≈ IC3 ≈ NA2 ≈NP3 ≈ IC2 ≈ IC1 > NP1 ≈ EU1 > NA1 (Figure 3).

#### 3.2.2. Gene x Environment Stress Tests

In Test 1, using a high temperature/low free-water stress environment (V82A with 2 % agar and incubation at 28.5 °C), nine random Vietnamese IC1 isolates were compared with three isolates each of the known *P. ramorum* lineages. Both NA2 and EU2 were highly tolerant (combined growth rate range 1.7–2.3 mm/d) and NA1 and EU1 highly intolerant (zero growth). In contrast, all IC1 isolates were weakly tolerant (range 0.3–0.6 mm/d), showing a different response from the known lineages. A second test at 28.5 °C (Test 2) included two isolates each of IC1 and IC2; the single IC3, IC4 and IC5 isolates; three NP1 isolates; two NP2 isolates; the single NP3 isolate and two isolates of each known lineages (Table 1). Again, EU2 and NA2 were highly tolerant, and EU1 and NA1 were highly intolerant (Table 1). IC1 and IC4 were also very tolerant (both 1.6 mm/d); IC3 was intermediate (0.8 mm/d), and both IC2 and IC5 were highly intolerant (zero growth). At 2.2 mm/d, the NP3 isolate showed high tolerance comparable to EU2 and NA2. NP2 was highly intolerant and failed to grow. NP1 was intermediate, but one NP1 isolate grew significantly faster (1.3 mm/d) than the other NP1 isolates (0.4–0.6 mm/d), indicating different genotypes within NP1.

**Figure 5:**
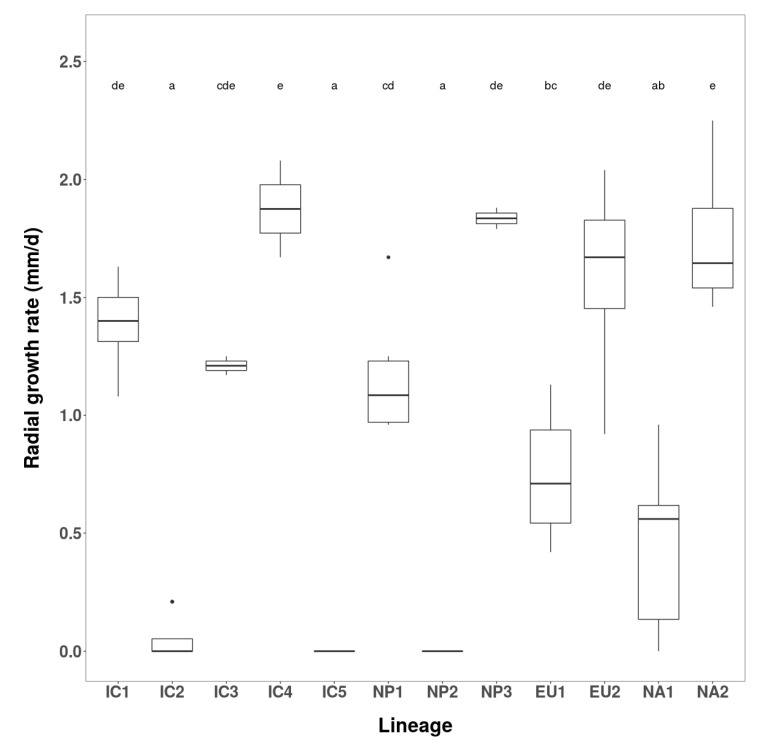
Box and whiskers diagram showing daily radial growth rates of the eight Asian phenotype groups and four known lineages of *Phytophthora ramorum* in the gene x environment stress test at 28 °C on 2% V8-agar (V82A). Different letters indicate statistical differences at significance level α = 0.05.

Test 3 was carried out at the marginally lower temperature 28 °C. Five IC1; four IC2; the single IC3, IC4 and IC5 isolates; three NP1 isolates; two NP2 isolates and the single NP3 isolate were again compared with the known lineages (Figure 5). The responses of known lineages were NA2 ≈ EU2 > EU1 > NA1 (Figure 5). The IC groups again exhibited a wide range of responses. IC4 isolate VN851 (1.9 mm/d) was again exceptionally tolerant, with similar growth to the EU2 and NA2 lineages. IC1 isolates, together with the single IC3 isolate (VN88), showed moderate to high tolerance (1.2–1.6 mm/d), while the IC2 isolates and the single IC5 isolate (VN863) were highly intolerant (0–0.2 mm/d) with growth below even the NA1 lineage (Figure 5). The NP groups also exhibited very different responses. The single NP3 isolate (JP975) had high tolerance (1.8 mm/d) comparable to IC4, EU2 and NA2; NP1 was moderately tolerant (1.1–1.3 mm/d) and NP2 failed to grow (Figure 5).

Overall, colony types could be used to distinguish the five IC and three NP phenotype groups from each other and from the known lineages. Additionally, growth rate tests at 20 °C and g x e tests at 28 and 28.5 °C revealed a range of adaptive differences in the phenotype groups similar to those of the four known lineages.

#### 3.2.3. Mating Tests

Forty-seven VN isolates were tested, initially using A1 and A2 tester isolates of the known lineages and later also Vietnamese isolates of known mating type. Gametangial frequency in each compatible pairing varied from very rare to scarce. Forty-two isolates (89%) were A1 and only five (11%) were A2. All 40 IC1 isolates tested were A1 as was the single IC4 isolate (Table 2). All four IC2 isolates and the single IC3 isolate were A2. Despite multiple mating tests the single IC5 isolate behaved as a functional non-responder or A0. Of the seven JP isolates, the four NP1 isolates from Shikoku and Kyushu were A1 whereas the two NP2 isolates and the single NP3 isolate were A2 (Table 2).

**Figure 6:**
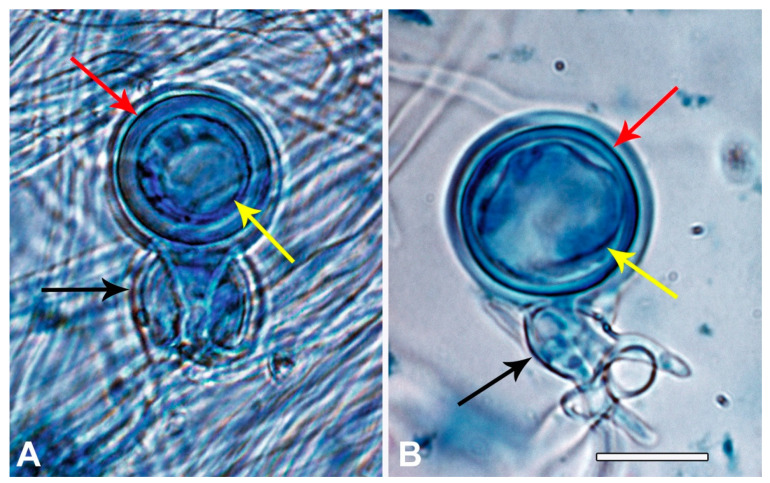
Oogonia from an IC1/A1 × IC2 /A2 pairing (isolates VN160 × VN314) with amphigynous antheridia (black arrows); structures stained with lactophenol blue. (**A**) Oogonium containing a viable oospore with a thick well-developed wall (red arrow), an oily granular matrix and a globose lipid vacuole (yellow arrow). (**B**) Oogonium containing an aborted oospore with a thin irregular wall (red arrow), a glassy refractive matrix with a disorganised vacuole (yellow arrow). Scale bar = 20 μm.

In multiple Vietnam IC1 × IC2 pairings gametangial form and size corresponded to the published descriptions for *P. ramorum* from EU1 × NA1 lineage pairings [38,79]. Mean oogonial diameter was 28.8 μm (range 25.0–32.5 μm), oospore diameter 26.1 μm (22.5–28.8 μm), oospore wall thickness 2.3 μm (1.25–2.5 μm), antheridial width 17.2 μm (15.0–20.0 μm) and antheridial length 15.8 μm (12.5–20.0 μm). Overall, 81.5 % of the IC1 × IC2 oogonia had visually normal oospores while 18.5 % were clearly aborted (Figure 6). In the Japanese pairing NP1 × NP2, however, many oospores appeared aborted, but gametangia were too scarce to estimate abortion rates.

### 3.3. Phylogenetic Analyses

Partial gene sequences of sixteen isolates representing the five IC phenotype groups and all seven Japanese isolates were obtained for seven nuclear and five mitochondrial genes and aligned with those from isolates of the four known *P. ramorum* lineages and two related *Phytophthora* species from Clade 8c, *P. lateralis* and *P. hibernalis*. Resulting lengths of amplified nuclear gene regions were *Avh120*, 387 bp; *Avh121*, 399 bp; *btub,* 899 bp; gwEuk.30.30.1, 682 bp; *hsp90*, 846 bp; ITS, 799 bp; and *trp1,* 818 bp; and of mitochondrial segments were *cox1*, 1199 bp; *cox2*, 905 bp; *nadh1*, 796 bp; Prv8, 344 bp; and Prv9, 518 bp. Within *P. ramorum,* the seven nuclear gene regions contained on average 2.8 % segregating sites (point mutations, heterozygous sites and indels), although their proportion varied considerably from 12.5% in *Avh121* to 0.6 % in ITS. The *Avh121* gene was most variable (12.5 %), indicating strong selection of this effector, followed by gwEuk.30.30.1 (4.4 %), *Avh120* (4.1 %), *trp1* (2.2 %); and the more conserved regions *btub* (1.0 %), *hsp90* (0.8 %) and ITS (0.6 %). Indels were only present in *Avh121* and gwEuk.30.30.1 (12 and 11 bp, respectively). In contrast, the five mitochondrial gene regions contained no heterozygous sites or indels and less segregating sites, averaging 1.2 % over all loci (range from 0.9 % in *nadh1* to 1.7 % in Prv8). Translation of nucleotide sequences into aminoacids showed that 81 of the 135 nuclear (60 %) and 42 of the 47 mitochondrial (89.4 %) segregating sites were silent (synonymous or in non-coding regions).

#### 3.3.1. Phylogenetic Trees

Separate phylogenetic analyses of all 12 loci using both Maximum Likelihood (ML) and Bayesian Inference (BI) could not resolve the phylogenetic positions of all IC and NP phenotypic groups and the known lineages due to scarcity of intraspecific signal. Indeed, some nuclear genes produced inconsistent and partly contradictory positions for the groups. To increase resolution, concatenated nuclear and mitochondrial datasets (4830 and 3762 bp, respectively) were analysed using the best fitted evolutionary model for each partition in the ML analysis and automatic model selection in the BI analysis.

**Figure 7:**
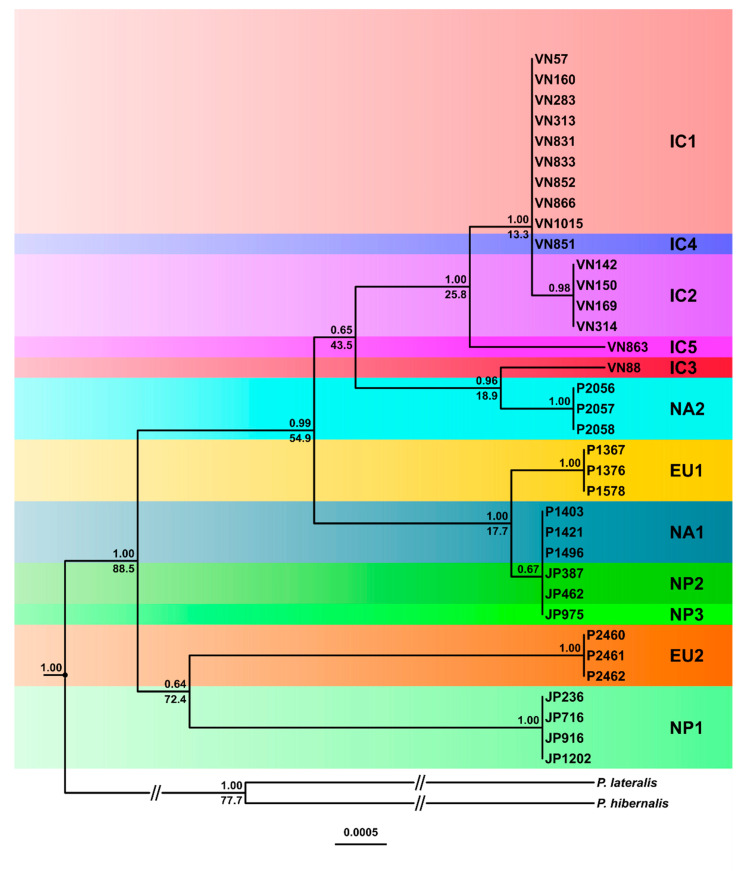
Fifty percent majority rule consensus tree derived from Bayesian inference analysis of a five-locus mitochondrial (*cox1*, *cox2*, *nadh1*, Prv8 and Prv9) dataset of the five Indochinese phenotype groups (IC1-5), the three Japanese phenotype groups (NP1-3) and the four known lineages (EU1, EU2, NA1 and NA2) of *Phytophthora ramorum*. Bayesian posterior probabilities and relative divergence times (in %) are indicated above and below branches, respectively. *Phytophthora hibernalis* and *P. lateralis* were outgroup taxa. Scale bar = 0.0005 expected changes per site per branch.

The BI and ML trees resulting from the concatenated mitochondrial dataset had some major differences, and posterior probability support in the BI analysis was higher than bootstrap support in the ML analysis (Figure 7 and Figure S3). In both analyses, IC4 resided within the nine IC1 isolates. Moreover, NA1, NP2 and NP3 shared identical mitochondrial sequences and clustered with high support in sister position to the EU1 lineage. All four IC2 isolates resided in sister position to IC1/IC4 while IC5 was basal to the cluster. EU2 and NP1 were also sister groups but clustered in different positions in the two analyses (Figure 7 and Figure S3). In the BI analysis, the EU2-NP1 cluster was basal to all other lineages, whereas in the ML analysis, it had a sister position to a large cluster comprising IC3, NA2 and the IC1-IC2-IC4-IC5 cluster (Figure 7 and Figure S3). In contrast, while the EU1-NA1-NP2-NP3 cluster was basal to all other lineages in the ML analysis (Figure S3), in the BI analysis, it resided in a sister position to a large cluster containing both the IC1-IC2-IC4-IC5 cluster and an NA2-IC3 cluster (Figure 7). NA2 and IC3 were also closely related in the ML analysis (Figure S3).

**Figure 8:**
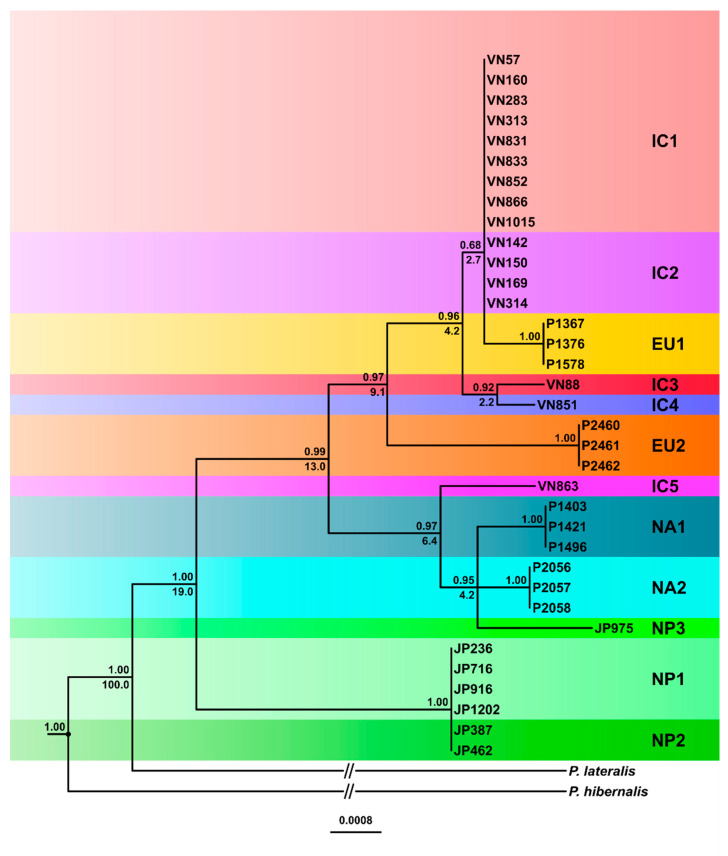
Fifty percent majority rule consensus tree phylogram derived from Bayesian inference analysis of a seven-locus nuclear (*Avh120*, *Avh121*, *btub*, gwEuk.30.30.1, *hsp90*, ITS and *trp1*) dataset of the five Indochinese phenotype groups (IC1-5), the three Japanese phenotype groups (NP1-3) and the four known lineages (EU1, EU2, NA1 and NA2) of *Phytophthora ramorum*. Bayesian posterior probabilities and relative divergence times (in %) are indicated above and below branches, respectively. *Phytophthora hibernalis* and *P. lateralis* were used as outgroup taxa. Scale bar = 0.0008 expected changes per site per branch.

With the concatenated nuclear dataset, the topologies of the BI and ML trees were similar although the BI analysis resolved the relative positions of more lineages (Figure 8 and Figure S4). However, neither analysis resolved the relative positions of NP1 and NP2, and IC1 and IC2, which differed across the 4830 bp alignment at 34 and eight heterozygous positions, respectively (Figure 8 and Figure S4). In the BI analysis, the NP1-NP2 cluster grouped with high support in sister position to the ancestor of the other ten lineages (Figure 8). In contrast, in the ML analysis, the highly supported basal node led to a polytomy of the three main clusters, IC1-IC2-IC3-IC4-EU1-EU2, IC5-NA1-NA2-NP3 and NP1-NP2 (Figure S4). IC3 and IC4 were sister groups residing in the BI analysis in a basal position to the IC1-IC2-EU1 cluster (Figure 8). In the ML analysis, the ancestor of IC3 and IC4 formed another polytomy with IC1/IC2 and the ancestor of EU1 (Figure S4). In both analyses, EU2 was basal to the large IC1 to IC4 and EU1 cluster, while IC5 was basal to the cluster containing NA1, NA2 and NP3. In the BI analysis NA1, NA2 and NP3 formed a highly supported polytomy whereas in the ML analysis NP3 was basal to NA1 and NA2 which formed together a weakly supported sister group (Figure 8 and Figure S4).

Overall, the phylogenetic trees showed that all eight Vietnamese and Japanese phenotype groups constituted separate evolutionary lineages. On that basis, they are redesignated as the IC1-5 and NP1-3 lineages. The main phenotypic and phylogenetic differences between all twelve lineages are summarised in Table S4.

#### 3.3.2. Phylogenetic Networks

**Figure 9:**
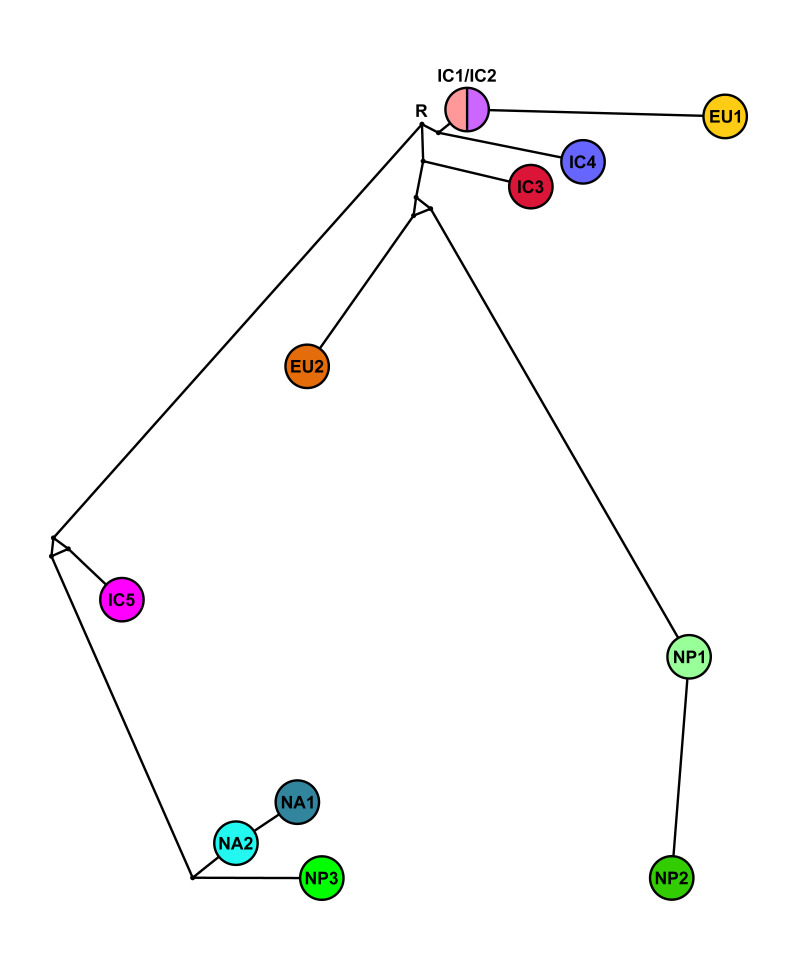
Median-joining network (MJN) with tolerance ε = 0 for the five Indochinese lineages (IC1-5), the three Japanese lineages (NP1-3) and the four known lineages (EU1, EU2, NA1 and NA2) of *Phytophthora ramorum* based on a seven-locus nuclear (*Avh120*, *Avh121*, *btub*, gwEuk.30.30.1, *hsp90*, ITS and *trp1*) dataset. The node with the highest number of connections and the most central position in this network and in the MJN with ε = 10 (Figure S5) was designated as root (R). Missing intermediates represent extant unsampled genotypes, extinct ancestors or “false-positives”. Branch lengths are proportional to the number of mutations.

**Figure 10:**
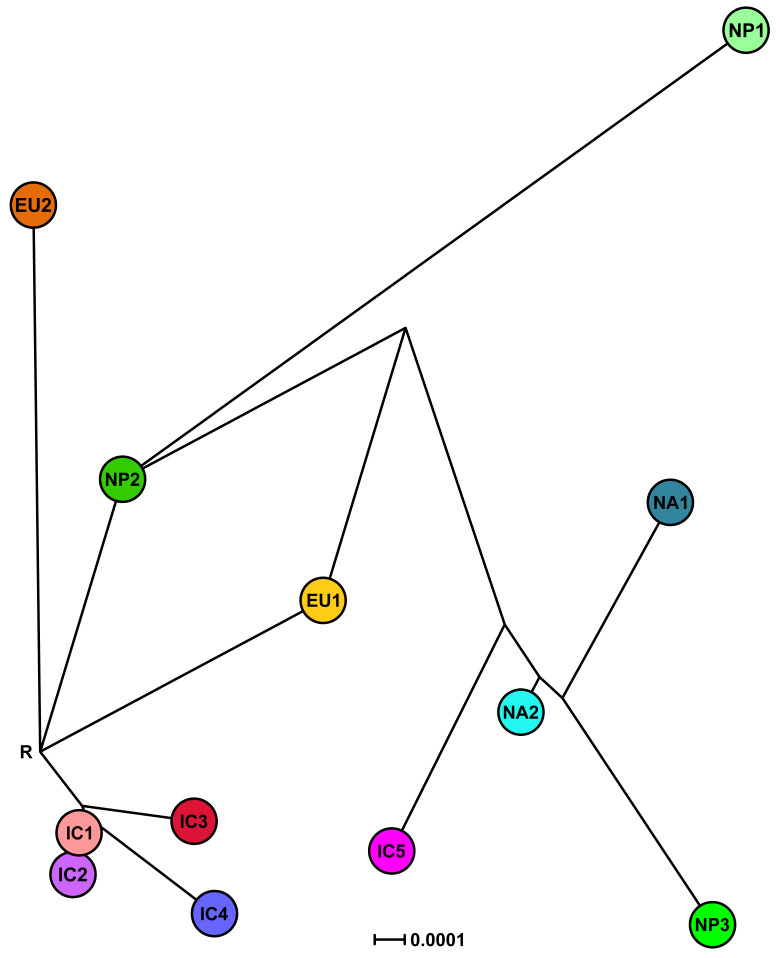
Split decomposition network (SDN) for the five Indochinese lineages (IC1-5), the three Japanese lineages (NP1-3) and the four known lineages (EU1, EU2, NA1 and NA2) of *Phytophthora ramorum* based on a seven-locus nuclear (*Avh120*, *Avh121*, *btub*, gwEuk.30.30.1, *hsp90*, ITS and *trp1*) dataset. Alternative pathways are shown as parallel edges. The node with the highest number of connections and the most central position was designated as root (R). Missing intermediates represent extant unsampled genotypes, extinct ancestors or “false-positives”. The scale bar represents the split support for the edges.

**Figure 11:**
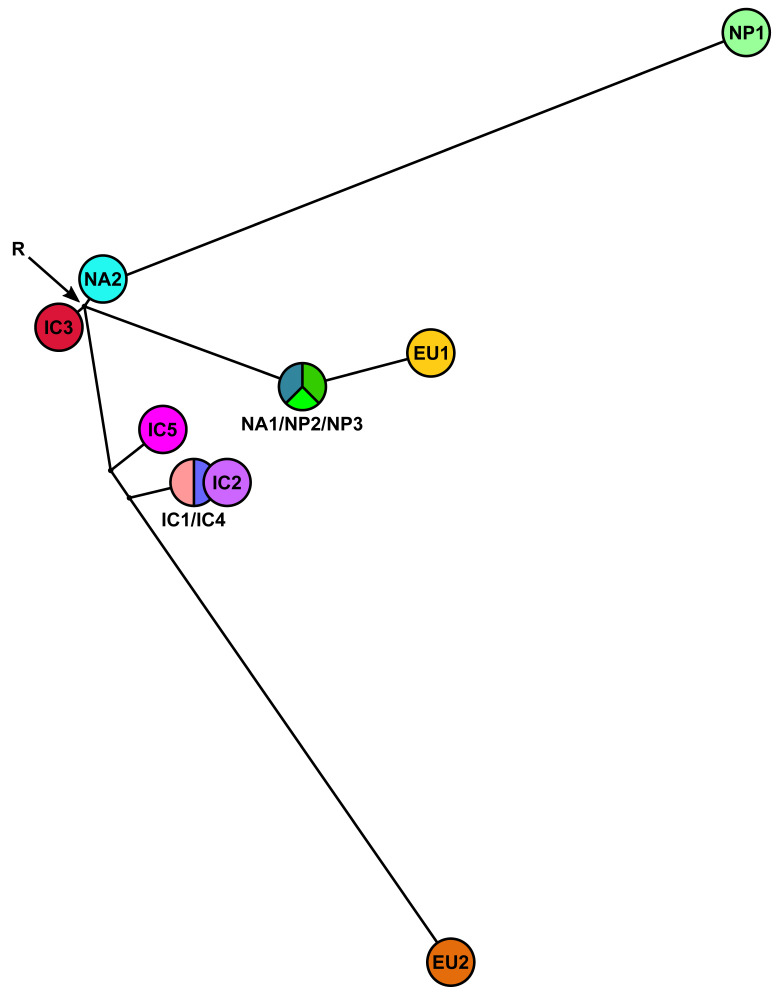
Median-joining network (MJN) with tolerance ε = 0 for the five Indochinese lineages (IC1-5), the three Japanese lineages (NP1-3) and the four known lineages (EU1, EU2, NA1 and NA2) of *Phytophthora ramorum*, based on a five-locus mitochondrial (*cox1*, *cox2*, *nadh1*, Prv8 and Prv9) dataset. The node with the highest number of connections and the most central position was designated as root (R). Missing intermediates represent extant unsampled genotypes, extinct ancestors or “false-positives”. Branch lengths are proportional to the number of mutations.

**Figure 12:**
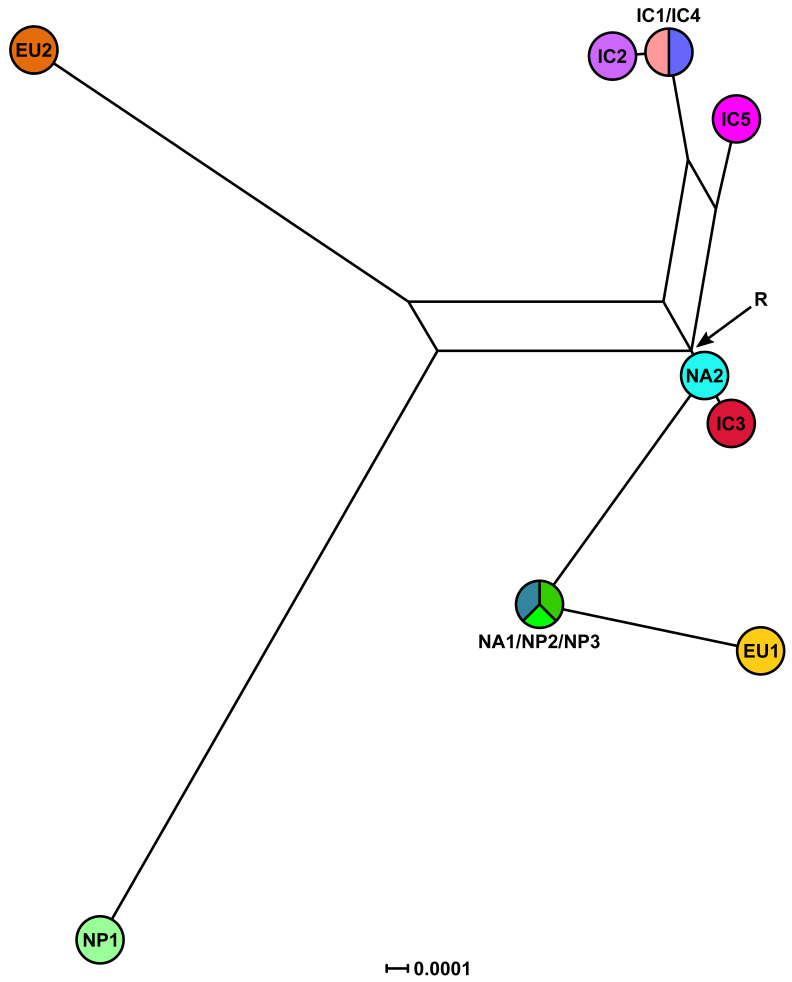
Split decomposition network (SDN) for the five Indochinese lineages (IC1-5), the three Japanese lineages (NP1-3) and the four known lineages (EU1, EU2, NA1 and NA2) of *Phytophthora ramorum* based on a five-locus mitochondrial (*cox1*, *cox2*, *nadh1*, Prv8 and Prv9) dataset. Alternative pathways are shown as parallel edges. The node with the highest number of connections and the most central position was designated as root (R). Missing intermediates represent extant unsampled genotypes, extinct ancestors or “false-positives”. The scale bar represents the split support for the edges.

To resolve character conflicts in the phylogenetic trees (indicated by low support values of several nodes, polytomies and zero length branches), we calculated median-joining networks (MJNs) and split decomposition networks (SDNs) for the concatenated datasets (Figure 9, Figure 10, Figure 11 and Figure 12, Figures S5 and S6). Both the MJNs and SDNs resolved most character conflicts. Nuclear MJNs with alternative tolerance settings of ε = 0 and ε = 10 both demonstrated that IC1 and IC2 were ancestors of EU1 with IC4 a sister group to the cluster (Figure 9 and Figure S5). NP1 was ancestral to NP2 and EU2 resided in sister position to NP1 and NP2 with IC3 diverging earlier from this cluster (Figure 9 and Figure S5). NA1 was an ancestor of NA2, with NP3 in sister position to NA1 and NA2, while IC5 diverged early from this cluster (Figure 9 and Figure S5). Increasing ε to 10 produced a MJN with the same basic structure as ε = 0 but with two additional median vectors, suggesting an alternative link for IC3 (Figure 9 and Figure S5).

While the nuclear MJNs did not resolve the positions of the IC1 and IC2 lineages, the nuclear SDN demonstrated that IC1 was ancestral to IC2 (Figure 10). EU1 emerged directly from the root and IC1, IC3 and IC4 radiated from the same unknown ancestor (Figure 10). EU2 and NP2 were also directly connected to the root. In contrast to the nuclear MJNs, in the nuclear SDN the ancestry of NP1 and NP2 was reversed (Figure 10). IC5, NA1, NA2 and NP3 had similar positions in both nuclear networks except that NA1 was not ancestral to NA2 in the SDN (Figure 9, Figure 10 and Figure S5).

In the mitochondrial MJN (ε = 0) the maternal lines of IC3 and NA2 emerged directly from the root (Figure 11) whereas in the mitochondrial trees both lineages had ambiguous positions (Figure 7 and Figure S3). The maternal line of NA1/NP2/NP3 also emerged directly from the root and was ancestral to EU1 (Figure 11). NP1 was a descendant of NA2 while EU2 resided at the opposite end of the network in sister position to IC1/IC4 and IC2 (Figure 11). The maternal line of IC1/IC4 was ancestral to IC2 (Figure 10). Increasing ε to 10 added more complexity close to the root including two additional median vectors (inferred genotypes) in the evolutionary history of EU2 (Figure S6). The mitochondrial MJNs and SDN shared main topological arrangements such as the direct emergence of NA2 from the root, the relative positions of IC1/IC4, IC2 and IC5 and the ancestral positions of IC1/IC4 to IC2 and of NA1/NP2/NP3 to EU1, respectively (Figure 11, Figure 12 and Figure S6). However, conversely to the mitochondrial MJNs, the mitochondrial SDN inferred NA2 was ancestral to IC3 and NA1/NP2/NP3 but not to NP1 (Figure 11, Figure 12 and Figure S6).

### 3.4. Inferred Evolutionary History of the IC, NP and Introduced Lineages

Based on the 81 silent substitutions across the seven nuclear loci and published average nuclear silent substitution rates we estimated the divergence between *P. ramorum* and *P. lateralis* at 2.4–8.4 million years (Myr) ago. Using the uncorrelated lognormal relaxed molecular clock model (allowing variable substitution rates along branches and between different branches of a phylogenetic tree), relative divergence times were estimated for the Bayesian nuclear and mitochondrial trees of the *P. ramorum* lineages. In the nuclear tree, the estimated relative time to the most recent common ancestor (TMRCA) was 19.0%, i.e., the earliest divergence between the most ancestral NP1 and NP2 lineages and the other ten lineages occurred after ca. 81% of the total evolutionary history of *P. ramorum* (Figure 8), between 456 Kyr and 1.6 Myr ago. At 13.0% relative time (312 Kyr–1.09 Myr) the IC5–NA1–NA2–NP3 cluster diverged from the cluster comprising IC1 to IC4, EU1 and EU2. Within the latter cluster, the EU2 separation from the other lineages was estimated at 9.1% relative time (218.4–764.4 Kyr), whereas in the former cluster IC5 diverged from the ancestor of NA1, NA2 and NP3 at 6.5% (156.0–546.0 Kyr). Intriguingly, major lineage divergences occurred in both clusters at 4.2% relative time (100.8–352.8 Kyr) between NA1, NA2 and NP3, and between the ancestor of EU1, IC1 and IC2 and the ancestor of IC3 and IC4 (Figure 8). IC3 and IC4 diverged relatively recently at 2.2% (52.8–184.8 Kyr); EU1 diverged from IC1 and IC2 at 2.7% (64.8–226.8 Kyr). The nuclear dataset lacked enough phylogenetic signal to calculate the divergence time of the closely related IC1 and IC2 lineages (Figure 8).

In general, the maternal mitochondrial lines (Figure 7) diverged much earlier than the nuclear lines, indicating a different evolutionary history. In the mitochondrial tree, the TMRCA of all twelve lineages was estimated at 88.5%, i.e., divergence between the ancestral maternal lines of EU2/NP1 and the other ten lineages had already occurred after 11.5% of the evolutionary history of *P. ramorum* (Figure 7). The EU2 and NP1 lineages also split early at 72.4% relative time, while EU1 and the shared maternal line of NA1, NP2 and NP3 diverged much later at 17.7%. Divergence between IC3 and NA2 and the other four IC lineages was estimated at 43.5% relative time, whereas divergence of IC3 and NA2 was much later (18.9%). Whilst IC5 split at 25.8% relative time from the maternal line leading to IC1/IC4 and IC2, the latter diverged only at 13.3% (Figure 7).

### 3.5. Distribution and Sympatry of Evolutionary Lineages and Mating Types Within Vietnam and Japan

The five IC lineages came from a small area of northern Vietnam (Figure 1, Table 2). All four IC2/A2 mating type isolates and the single IC3/A2 isolate were obtained from two closely adjacent Fansipan Streams, S3 and S4 (Table 2 and Table S1). Both streams, together with Streams S5 and S7, are mid altitude (ca 1900 m asl) locations that are environmentally disturbed due to heavy regional tourist impact. A single isolate of the common IC1 /A1 lineage was also obtained from Stream S4. The samples from Streams S3 and S4, therefore, demonstrated a highly localised distribution of IC2 and IC3, close local overlap (sympatry) of three of the five IC lineages, and close sympatry of the lineage-specific A1 and A2 mating types. The IC1 /A1 lineage dominated Fansipan Streams S1, S2, S5, S7 and S10 (Figure 1; Table 2), comprising 40 of the 41 isolates. Streams S1 and S2 are more remote, slightly higher altitude (2000–2100 m asl) catchments with less human disturbance. Low altitude Stream S10 (1190 m asl) is more distant from the other Fansipan streams (Figure 1) and originates from mostly undisturbed forests. The single IC5 /A0 isolate was also obtained from Stream S1, demonstrating close sympatry of two phylogenetically divergent lineages, IC1 and IC5. The single IC4 /A1 isolate was the only *P. ramorum* isolate obtained from the more distant Stream S11 (1300 m asl), a catchment on Sau Chua Mountain near Sin Chai village, 14 km northeast of the Fansipan catchments (Figure 1).

In the Japanese samples, the three NP lineages were mostly geographically separated. Three of the four NP1/A1 isolates came from an almost undisturbed stream catchment S15 near the top of a remote mountain in the Shimanto area on Shikoku island (Figure 1; Table 2 and Table S1). The two NP2/A2 isolates and the NP3/A2 isolate came from different stream catchments with limited disturbance, S22 and S31 respectively, on Kyushu island. A fourth NP1/A1 isolate, JP1202, also came from S31. Japanese samples, therefore, also demonstrated highly local overlap of evolutionarily divergent lineages and of different mating types.

## 4. Discussion

We have shown that populations of *P. ramorum* are present in the species-rich laurosilva forests around Fansipan and Sau Chua Mountains along the Vietnam–China border and on Shikoku and Kyushu islands in southwest Japan. Several lines of evidence indicate that these populations are native. First, in contrast to the introduced *P. ramorum* lineages in Europe and North America, these east Asian populations are very diverse. Despite the small, highly localised samples, multiple phenotypically and phylogenetically different lineages were present in both areas. Second, unlike the introduced lineages, the A1 and A2 mating types co-occur in these Asian populations. Third, despite the presence of many potential hosts in the Lauraceae, Fagaceae and Ericaceae, we saw no overt host symptoms in any sampled area, consistent with the view that native Phytophthoras cause limited damage to their co-evolved hosts [1,3,4,6,80,81]. Nonetheless, *P. ramorum* was isolated readily from detached leaves and flowers that had fallen into forest streams or onto the forest floor. Fourth, at the Shikoku and Kyushu sites, we also isolated two of *P. ramorum*’s three closest phylogenetic relatives in *Phytophthora* Clade 8c, namely *P. foliorum* and *P. lateralis* (Table S1). Like *P. ramorum,* these are damaging introduced pathogens in Europe and North America [4,8,28,82,83,84,85]. Overall, we conclude that northern Indochina, meaning Sau Chua mountain and the Hoàng Liên mountain range, which stretches from north Vietnam to the Ailao Shan range in Yunnan [86] and Shikoku and Kyushu in south west Japan, lie within the centre of origin of *P. ramorum*.

Indeed, the co-occurrence of *P. ramorum*, *P. foliorum* and *P. lateralis* in the Japanese laurosilva forests suggests that south west Japan could be the primary origin of *P. ramorum* and possibly of Clade 8c as a whole. An arc of montane laurosilva forests with similar genera and species occurs from east Myanmar through northern Laos, northern Vietnam and southern China to Japan [86,87,88,89,90,91,92]. Intriguingly, the flora of southeastern Yunnan and northern Vietnam was mainly derived from the South China Geoblock and is closely related to the East Asian flora [80,93]. It is likely that *P. ramorum* is native throughout this ecosystem and that additional lineages occur. Outlier laurosilva communities occur as far west as the Nepal–Bhutan border, Taiwan, and in the Malay Peninsula and the Greater Sunda Islands [94,95,96,97]. Past surveys for *P. ramorum* in Nepal and Taiwan have been negative [24,25,27], nor has the pathogen been reported from northern Yunnan or Hainan island east of the Vietnamese Gulf of Tonkin [26,98]. Nonetheless, *P. ramorum* may occur naturally at some of these other locations.

Taking into account *P. ramorum*’s dependence on rain and fog events for dispersal and infection and its etiology on infected larch, rhododendron and California bay laurel in Europe and North America [99,100,101,102,103,104,105], we suggest in the Asian laurosilva forests *P. ramorum* may specialise in colonising seasonally juvenile and senescing foliage during the monsoon rains, with new infections initiated by inoculum from quiescent infections on attached leaves or from the leaf litter. As demonstrated by our isolations, *P. ramorum* is also a successful coloniser of freshly fallen leaves in the forest streams. In North America, both aquatic and aerial populations of *P. ramorum* have a similar genetic structure and temporal pattern [106]. Therefore, the predominantly aquatic Asian *P. ramorum* populations we sampled are also likely to be representative of the aerial populations.

Despite some genealogical incongruences, the majority of nodes in all phylogenetic trees generated by the ML and BI analyses had strong bootstrap and posterior probability support, clearly demonstrating that the eight Asian phenotype groups constituted distinct phylogenetic lineages. Incongruences between ML and BI analyses result from differences in their algorithms [107] whereas discordances between mitochondrial and nuclear genealogies usually reflect incomplete lineage sorting or mitochondrial introgression [108,109,110,111]. Since, in the present study, both the individual mitochondrial and nuclear gene trees were incongruent with the concatenated multilocus trees, the mitonuclear discordance almost certainly resulted from incomplete lineage sorting [109,112]. However, the phylogenetic networks resolved most conflicts in both the ML and BI concatenated datasets. The twelve lineages most probably constitute extant ancestors (e.g., the IC1 and NA2 lineages in both MJNs and in the mitochondrial SDN) and their direct or indirect descendants (e.g., EU1 in both MJNs and IC2, NA1, NA2 and NP3 in both SDNs). To establish whether the missing intermediates are unsampled extant genotypes, extinct ancestors or “false positives” additional surveys across unsampled regions of east Asia are needed. As in the phylogenetic trees, the mitochondrial and nuclear networks (MJNs and SDNs) differed in their topologies, confirming different evolutionary histories for these two genomic regions. Moreover, the MJNs and SDNs were not fully congruent. Currently, therefore, the true phylogenetic relationships among what are now twelve lineages of *P. ramorum* remain largely ambiguous. Microsatellite and whole-genome-analyses are in progress to further resolve their relationships and migration patterns.

The macro- and microevolutionary processes underlying the considerable lineage diversity in *P. ramorum* also remain unclear. The EU1, EU2, NA1 and NA2 lineages introduced into Europe and North America were already believed to be ancient, with the EU2 lineage estimated to have diverged first >1 million years ago [15,16,18]. Based on silent substitutions across nuclear genes and published average nuclear substitution rates, we dated the time to the most recent common ancestor of *P. ramorum* and its closest relative *P. lateralis* at 2.4–8.4 Myr, slightly earlier than the 1.5–5.4 Myr estimated by Goss et al. [15] but similar to the 5.9–6.8 Myr estimated by Dale et al. [18] who calibrated their molecular clock analyses with divergence time estimates from the strict clock analysis of Matari and Blair [113]. From our nuclear coalescence analysis, we estimated that the twelve lineages of *P. ramorum* shared 81% of their evolutionary history and that the Japanese NP1/NP2 lineages diverged first at between 0.5 and 1.6 Myr ago. The splitting of the NA1, NA2 and NP3 lineages and the divergence between the ancestors of the IC3-IC4 and EU1-IC1-IC2 clusters occurred near simultaneously suggesting a major selection event at 4.2% relative evolutionary time (100.8–352.8 kyr), probably related to climatic cooling and drying during a pleistocenic glaciation period (discussed in next paragraph). It is noteworthy that, despite its current co-occurrence with the other four Indochina lineages in a very small area of northern Vietnam, the IC5 lineage may have diverged from them ca 0.3 to 1.1 Myr ago. The divergence of the maternal lines, however, occurred much earlier in the evolutionary history of *P. ramorum*, with the maternal ancestor of EU2 and NP1 having already split from the other lineages at 11.5% relative time. This intraspecific retention of anciently separated maternal lines indicates long- term isolation of *P. ramorum* populations, extending coalescence times far beyond those observed in species with a single panmictic population [114,115].

In addition to their often considerable phylogenetic divergence, the twelve lineages exhibit differences in colony morphology, adaptive behaviour (summarised in Table S4) and, in the case of the four known lineages (EU1, EU2, NA1 and NA2), pathogenicity to forest trees comparable to those usually seen between different *Phytophthora* species [20,21,22,116], paralleling similar differences between lineages of *P. lateralis* [29]. Therefore, it appears most likely that the lineages have evolved from a common ancestor through geographic isolation, local adaptation and drift. This is also consistent with the view that alternations across East Asia between long, cool and dry glacial periods and shorter, warmer and more humid interglacial periods caused repeated retreats into isolated refugia, resulting in high and disjunct post-Pleistocene radiation and species diversity [92,117,118,119,120]. This process was particularly pronounced in northern Indochina [93,121,122,123,124] where dissection of the Yunnan plateau and north Vietnam by high mountain ranges and deep river valleys created “mountain islands” [89,125]. The Japanese islands were repeatedly connected with each other and mainland Asia during interglacial periods via temporary land bridges that facilitated migrations followed by isolation and speciation processes during subsequent glacial periods [87,120].

However, in our phylogenetic analyses, the eight additional lineages of *P. ramorum* discovered in northern Indochina and Japan are scattered across the topology of the four known lineages. Furthermore, phylogenetically and adaptively distinct lineages co-occur both in the Fansipan Mountain area (e.g., IC1, IC3 and IC5) and on Kyushu island (NP1 and NP3). A long-term coexistence of such divergent lineages might be accounted for through their being strongly reproductively isolated and adapted to different hosts or other ecological niches. There is some evidence to support this hypothesis, since the EU1 and NA1 lineages exhibit a degree of pre-and postzygotic reproductive isolation, discussed below. Nonetheless, we favour the view that the co-occurrence of highly divergent lineages at the Fansipan and on Kyushu is most likely due to recent, human-mediated spread of previously geographically isolated lineages, e.g., via chlamydospores in soil adhering to hikers boots or vehicles. Our evidence that the NP2 and NP3 lineages have near identical mitochondrial gene sequences to NA1 and EU1, respectively, but differ significantly in their nuclear genes, suggests their respective maternal ancestors may once have had a widespread distribution but subsequently outcrossed with rather different paternal genotypes. This also applies to the IC3 and NA2 lineages.

Another outstanding issue is the role of sexual reproduction in current lineage microevolution. In Europe and North America the introduced *P. ramorum* lineages each comprise only a single mating type and oospores have not been observed in naturally infected plant tissues. A1 × A2 matings between them are typically of low fertility, the oospores frequently abort, and the progeny of EU1 × NA1 matings are unfit, indicating both pre- and post-zygotic isolation [36,38,126]. While the more frequently found lineages described here, IC1, IC2 and NP1, were also of a single mating type, this might simply reflect local founder effects and the small sample sizes. We also found for the first time local co-occurrence of the A1 and A2 mating types, albeit belonging to separate lineages. Indeed, the locally co-occurring, phenotypically distinct IC1 (A1) and IC2 (A2) lineages were almost identical in their mitochondrial gene sequences and differed in their nuclear gene sequences by only a few heterozygous positions. Further, in matings between them we observed only a low level of oospore abortion, suggesting good genomic congruity and the possibility of some sexual outcrossing in at least the Fansipan population. The failure of the single IC5 lineage isolate to mate with either A1 or A2 compatibility type testers might reflect accumulation of deleterious mutations in genes involved in gametogenesis [127] following long-term geographic isolation from the other IC lineages, or result from sterility due to recent, aberrant sexual or somatic recombination. To resolve such issues more detailed investigation of gene flow in the Indochina and Japanese populations, including inter-lineage crosses, is needed.

Aspects of asexual reproduction will also have driven the observed variation. Under epidemic conditions in European and North American forests *P. ramorum* often spreads exponentially and clonally, via asexual sporangia, from foliage infection to foliage infection across the canopy. Therefore the 47 isolates of the locally predominant Fansipan IC1 lineage, which were all A1 mating type and shared the same colony type and similar adaptive behaviour, probably also spread initially as an asexual clone. The IC1 isolates did however have significantly different growth rates. Hence, over time IC1 has probably accumulated genetic changes via mutation or genome rearrangement, as already well documented for the introduced lineages [13,17,18,19,20,128,129]. The same may also apply to the IC2 lineage. Through these asexual processes, better adapted genotypes or genotypes that establish earlier in a season can become dominant [13,106]. Thus the dominant, pan-European genotype of the EU1 lineage currently epidemic on larch in Britain is a more prolific sporulator than otherwise unique British EU1 genotypes from woody ornamentals and broadleaved trees [13]. The spatial distribution of susceptible hosts (still an unknown variable) across the laurosilva forest canopy, and local climatic events [130], will also be influential. The local predominance of IC1 may reflect a combination of these processes, and the overall heterogeneity of the Indochinese and Japanese *P. ramorum* samples may be just a momentary snapshot of a locally changing pattern of scarcer and more dominant lineages, and possibly also recombinants.

One of the main objectives of this work was to try to identify the geographic origins of *P. ramorum*. A future objective must be to elucidate its “true” hosts and behaviours in its native habitats. Another objective of this work was to better understand the scale of the biosecurity threat posed to the world’s forests by the growing international trade in living plants. This trade is a major conduit for alien pests and pathogens [1,2,3,4,131,132,133]. However current international plant health (“SPS”) protocols, based on World Trade Organisation rules, allow only named threat organisms to be proscribed [1]. Hence, there is a substantial information gap between these protocols and the potentially enormous number of undescribed threat organisms, putting the world forests at serious risk of further epidemics. Phytophthoras, arguably the world’s most infamous and damaging plant pathogens, are particularly well adapted to spread via the international nursery trade [4,134], and it has been estimated that there may be as many as 400–500 undescribed *Phytophthora* species worldwide [7]. Recent surveys in Taiwan and Vietnam forests alone have identified 38 previously unknown *Phytophthora* species [27,30,33]. Many developing countries, often with natural ecosystems largely unexplored for forest pathogens, are gearing up to produce horticultural plants for export. To better understand the scale of the threats, there is an urgent need for further surveys in underexplored forest ecosystems and for extensive host range testing of previously unknown *Phytophthora* species and lineages.

## 5. Conclusions

Our research indicates that the destructive, introduced Oomycete pathogen *P. ramorum*, currently epidemic on larch in parts of Europe and oaks and tanoaks in California and Oregon, probably originates from the laurosilva forests between Indochina and south west Japan. Combined phenotypic and phylogenetic characterisation of the isolates sampled shows that multiple ancient and differently adapted evolutionary lineages are present in the area which probably started to diverge between 0.5 and 1.6 Myr ago. Lineage evolution has likely been driven by repeated glaciations and geographic isolation. Local co-occurrence of lineages of “opposite” mating type indicates the potential for inter-lineage sexual recombination. Our findings emphasize the substantial threat posed to forests worldwide by introductions of previously “unknown” pathogens from underexplored ecosystems.

## Figures and Tables

**Table 1:** Growth x environment stress responses of the Indochinese (IC) and Japanese (NP) phenotype groups and the known lineages of *Phytophthora ramorum* on 2% V8-juice agar at 28.5 °C.

Phenotype Group	Isolates		Radial Growth (mm/d) over 12 Days	
	No.	Codes	Range	Mean
Vietnam				
IC1	2	VN160, VN856	1.5–1.63	1.56
IC2	2	VN142, VN150	0	0.0
IC3	1	VN88	-	0.82
IC4	1	VN851	-	1.63
IC5	1	VN863	-	0.0
Japan				
NP1	3	JP236, JP718, JP1202	0.44–1.25	0.76
NP2	2	JP387, JP462	0	0.0
NP3	1	JP975	-	2.15
Known lineages				
EU1	2	P1367, P1376	0–0.42	0.21
EU2	2	P2460, P2461	2.33–2.46	2.39
NA1	2	P1403, P1421	0	0.0
NA2	2	P2056, P2058	2.21–2.46	2.33

-, Not available.

**Table 2:** Distribution of phenotype groups and mating types of *Phytophthora ramorum* across stream catchments in Vietnam and Japan.

Region (Altitude (m asl))	Stream Number ^1^	Phenotype Group ^2^	No. of Isolates by Mating Type		
			A1	A2	A0 ^3^
Vietnam (VN)					
Fansipan (2083)	S1	IC1 IC5	16		1
Fansipan (2007)	S2	IC1	9		
Fansipan (1913)	S3	IC2		4	
Fansipan (1904)	S4	IC1 IC3	1	1	
Fansipan (1895)	S5	IC1	3		
Fansipan (1912)	S7	IC1	7		
Fansipan (1193)	S10	IC1	4		
Sau-Chua (1308)	S11	IC4	1		
Japan (JP)					
Shikoku, Shimanto (523)	S15	NP1	3		
Kyushu, Tarumizu (546)	S22	NP2		2	
Kyushu, Kirishima (335)	S31	NP1 NP3	1	1	

^1^ For geographical locations, see Figure 1 and Table S1; S, regional stream catchment number. ^2^ IC, Indochina; NP, Nippon = Japan. ^3^ A0, functional non-responder.

## Data Availability

All sequences generated during this study are available from GenBank and accession numbers are given in Table S2. All datasets and trees deriving from BI and ML analyses are available from TreeBASE (ID 27745; www.treebase.org/ (accessed on 15 March 2021)).

## Supplementary Materials

The following are available online at https://www.mdpi.com/2309-608X/7/3/226/s1. Table S1. Location, altitude and vegetation of the forest stream catchments in northern Vietnam and Japan included in the *Phytophthora ramorum* survey, sampling method, lineages, mating types (A1, A2, A0, n.t. = not tested) and isolate codes. Table S2. GenBank accession numbers of seven nuclear and five mitochondrial gene regions generated in this study for 35 isolates from 12 lineages of *Phytophthora ramorum* and one isolate of *P. lateralis* and whole genome shotgun sequence contig numbers of GenBank assembly accession GCA_012656075.1 for *P. hibernalis* isolate BL41G. Table S3. Details of primers, annealing temperatures and Mg^2+^ concentrations used to amplify the genomic regions investigated in this study. Table S4. Summary of main traits discriminating the 12 *Phytophthora ramorum* lineages from Indochina (IC), Japan (NP), Europe (EU) and North America (NA). Figure S1. Box and whiskers diagram showing daily radial growth rates of 50 isolates from the IC1 phenotype group of *Phytophthora ramorum* at 20 °C on carrot agar (CA). Figure S2. Box and whiskers diagram showing daily radial growth rates of three isolates from the IC2 phenotype group of *Phytophthora ramorum* at 20 °C on carrot agar (CA). Figure S3. Fifty percent majority rule consensus phylogram derived from Maximum Likelihood (ML) analysis of a mitochondrial five–loci (*cox1*, *cox2*, *nadh1*, Prv8 and Prv9) dataset of the five Indochinese phenotype groups (IC1-5), the three Japanese phenotype groups (NP1-3) and the four known lineages (EU1, EU2, NA1 and NA2) of *Phytophthora ramorum*. Figure S4. Fifty percent majority rule consensus phylogram derived from Maximum Likelihood (ML) analysis of a nuclear seven–loci (*Avh120*, *Avh121*, *btub*, gwEuk.30.30.1, *hsp90*, ITS and *trp1*) dataset of the five Indochinese phenotype groups (IC1-5), the three Japanese phenotype groups (NP1-3) and the four known lineages (EU1, EU2, NA1and NA2) of *Phytophthora ramorum*. Figure S5. Median-joining network (MJN) with tolerance ε = 10 for the five Indochinese lineages (IC1–IC5), the three Japanese lineages (NP1–NP3) and four known introduced lineages (EU1, EU2, NA1 and NA2) of *Phytophthora ramorum* based on a nuclear seven–loci (*Avh120*, *Avh121*, *btub*, gwEuk.30.30.1, *hsp90*, ITS and *trp1*) dataset. Figure S6. Median-joining network (MJN) with tolerance ε = 10 for the five Indochinese lineages (IC1–IC5), the three Japanese lineages (NP1–NP3) and the four known lineages (EU1, EU2, NA1 and NA2) of *Phytophthora ramorum* based on a mitochondrial five–loci (*cox1*, *cox2*, *nadh1*, Prv8 and Prv9) dataset.
