# Supplementary material for: The Destructive Tree Pathogen Phytophthora ramorum Originates from the Laurosilva Forests of East Asia
Source: J Fungi (Basel). 2021 Mar 18;7(3):226. doi: 10.3390/jof7030226 (PMC8003361; doi:10.3390/jof7030226)
Supplement: Supplementary file 1 [file jof-07-00226-s001.zip › Jung et al. 2021_Supplementary Figures S1-S6.pdf]

Supplementary Figures

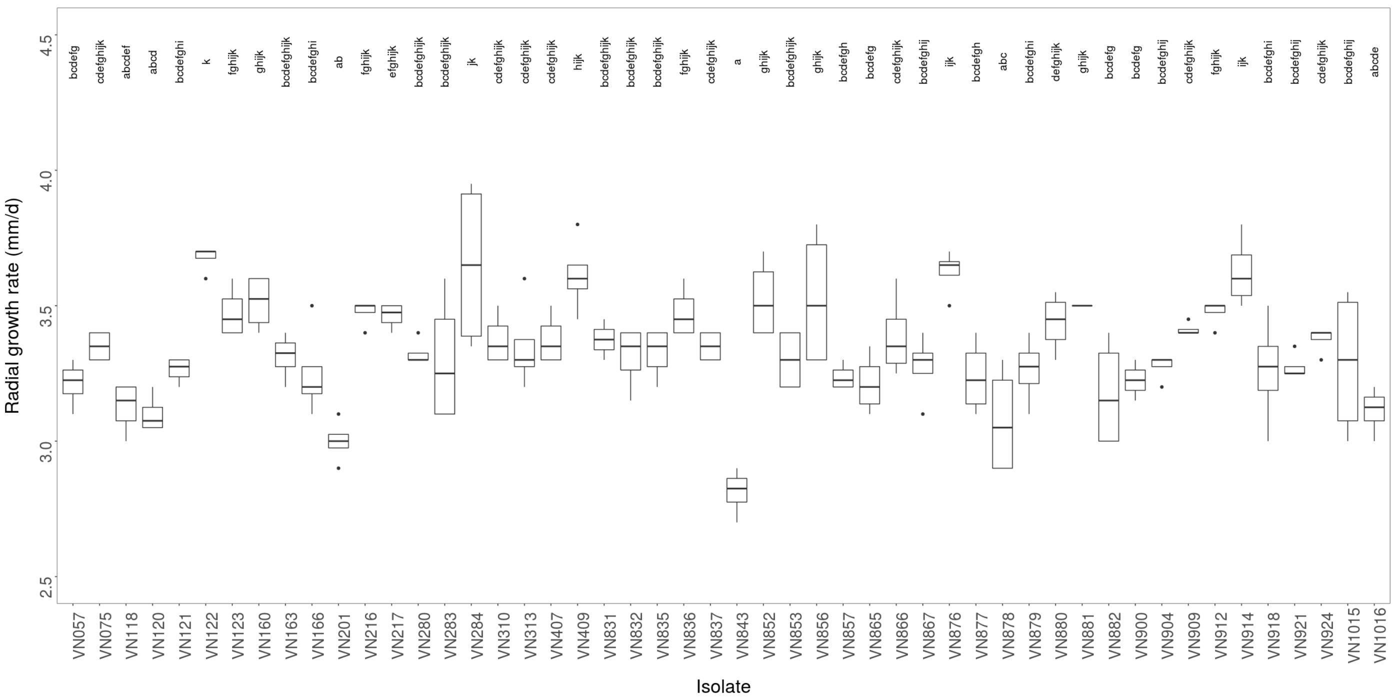

Supplementary Figure S1. Box and whiskers diagram showing daily radial growth rates of 50 isolates from the IC1 phenotype group of *Phytophthora ramorum* at 20 °C on carrot agar (CA). Different letters indicate statistical differences at significance level α = 0.05.

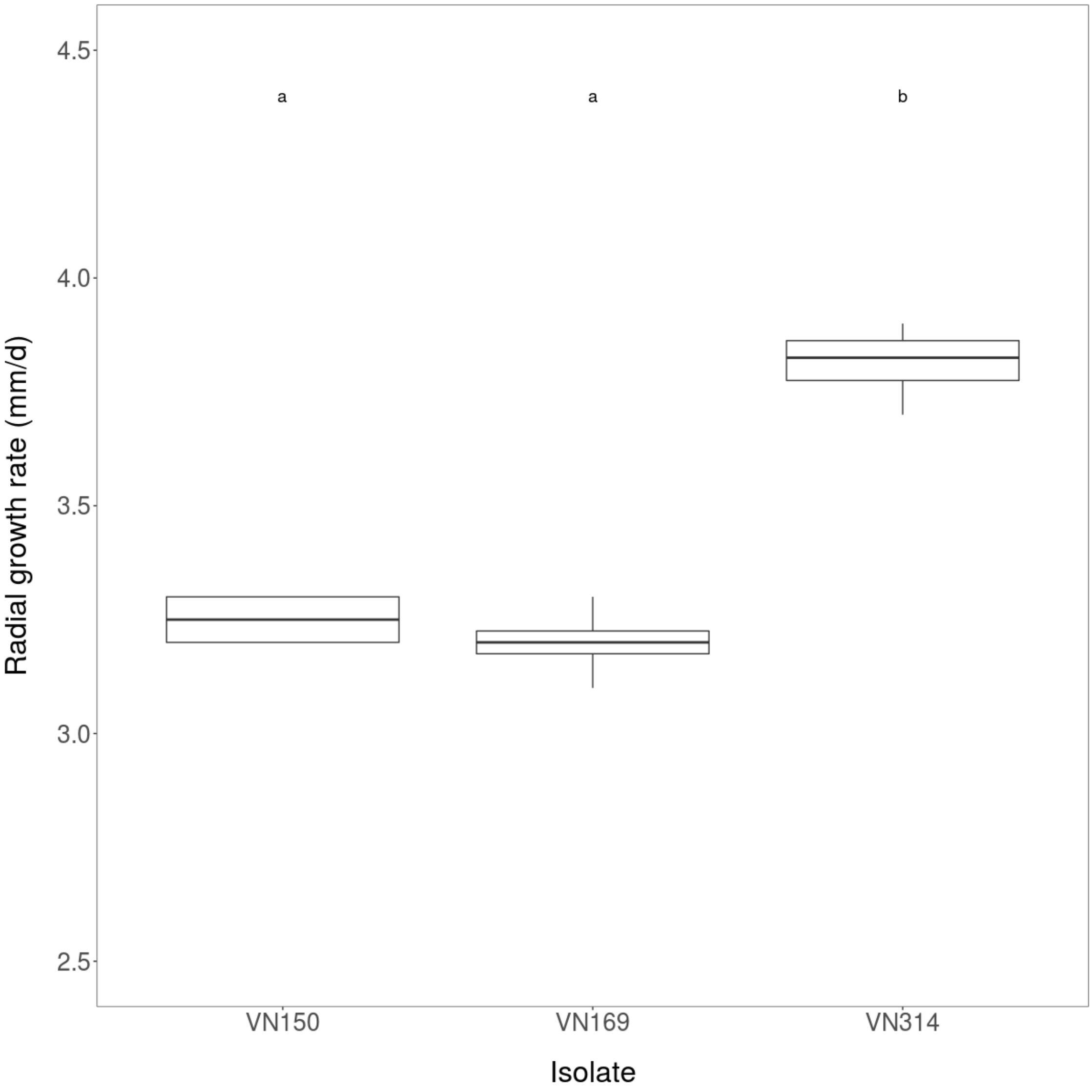

Supplementary Figure S2. Box and whiskers diagram showing daily radial growth rates of three isolates from the IC2 phenotype group of *Phytophthora ramorum* at 20 °C on carrot agar (CA). Different letters indicate statistical differences at significance level α = 0.05.

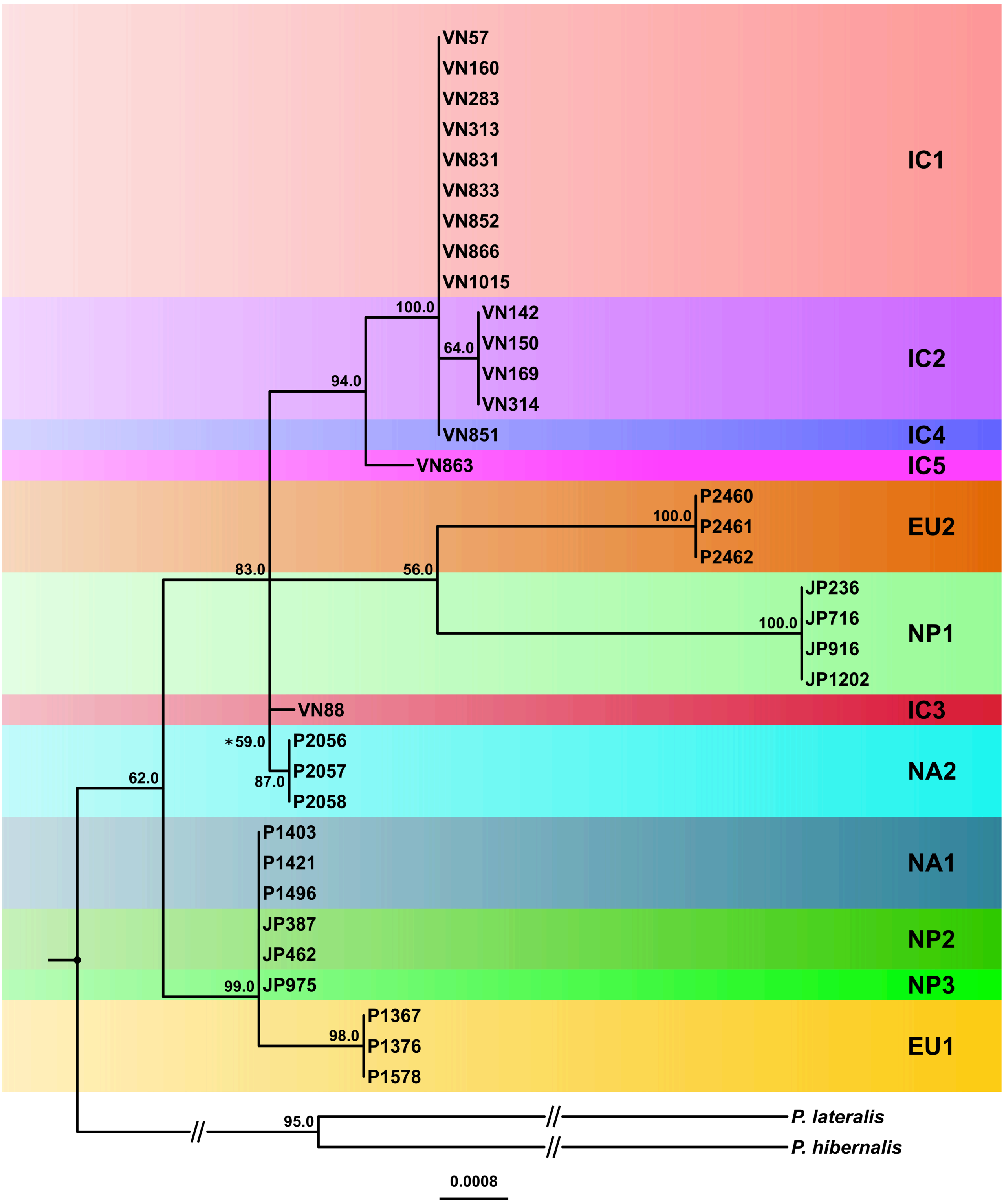

Supplementary Figure S3. Fifty percent majority rule consensus phylogram derived from Maximum Likelihood (ML) analysis of a mitochondrial five–loci (*cox1*, *cox2*, *nadh1*, Prv8, Prv9) dataset of the five Indochinese phenotype groups (IC1-5), the three Japanese phenotype groups (NP1-3) and the four known lineages (EU1, EU2, NA1, NA2) of *Phytophthora ramorum*. Maximum Likelihood bootstrap values (in %) are indicated above branches. *Phytophthora hibernalis and P. lateralis* were used as outgroup taxa. Scale bar = 0.0008 expected changes per site per branch. * Invisible node due to extremely short branch length.

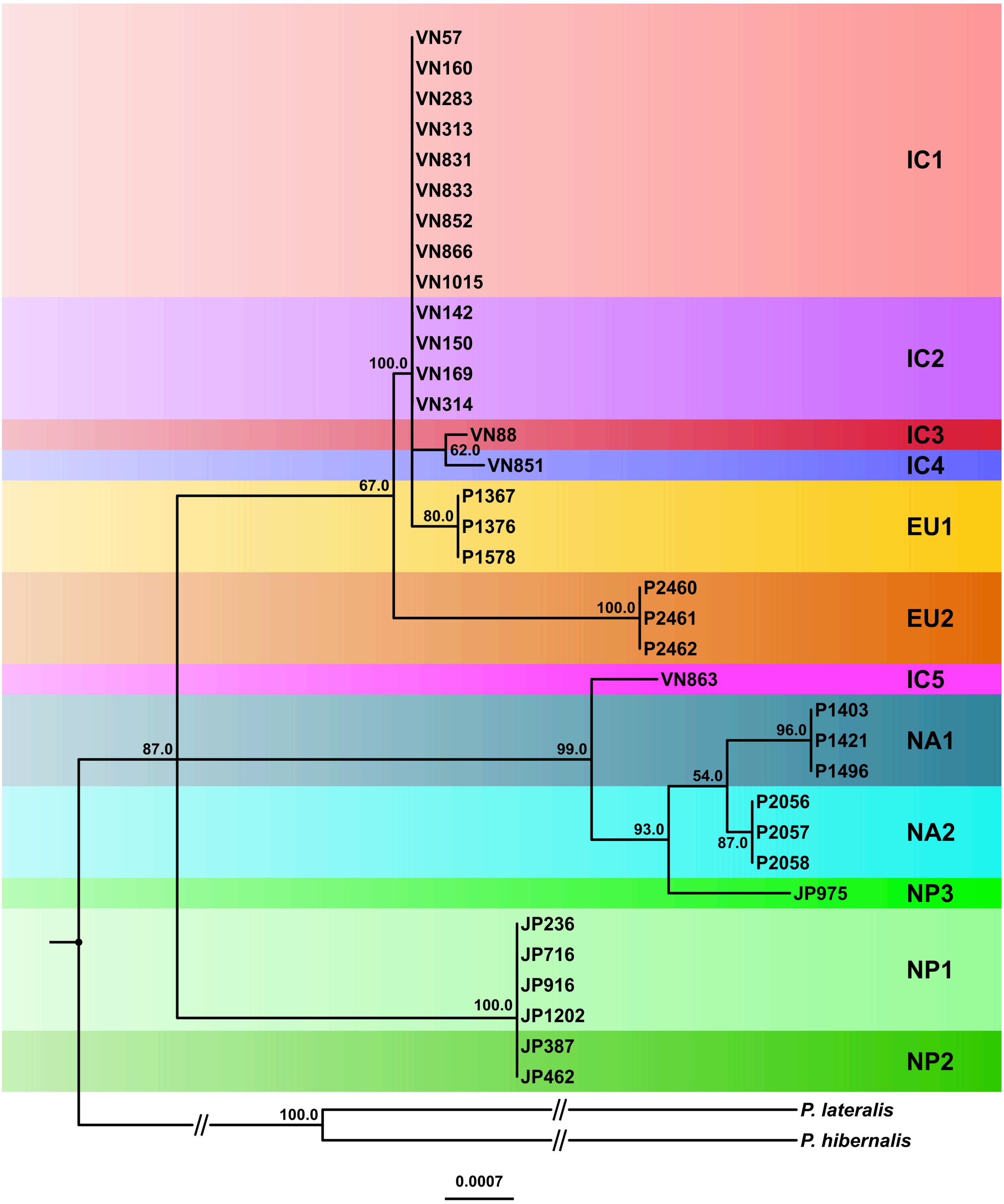

Supplementary Figure S4. Fifty percent majority rule consensus phylogram derived from Maximum Likelihood (ML) analysis of a nuclear seven–loci (*Avh120*, *Avh121*, *btub*, gwEuk.30.30.1, *hsp90*, ITS, *trp1*) dataset of the five Indochinese phenotype groups (IC1-5), the three Japanese phenotype groups (NP1-3) and the four known lineages (EU1, EU2, NA1, NA2) of *Phytophthora ramorum*. Maximum Likelihood bootstrap values (in %) are indicated above branches. *Phytophthora hibernalis and P. lateralis* were used as outgroup taxa. Scale bar = 0.0007 expected changes per site per branch.

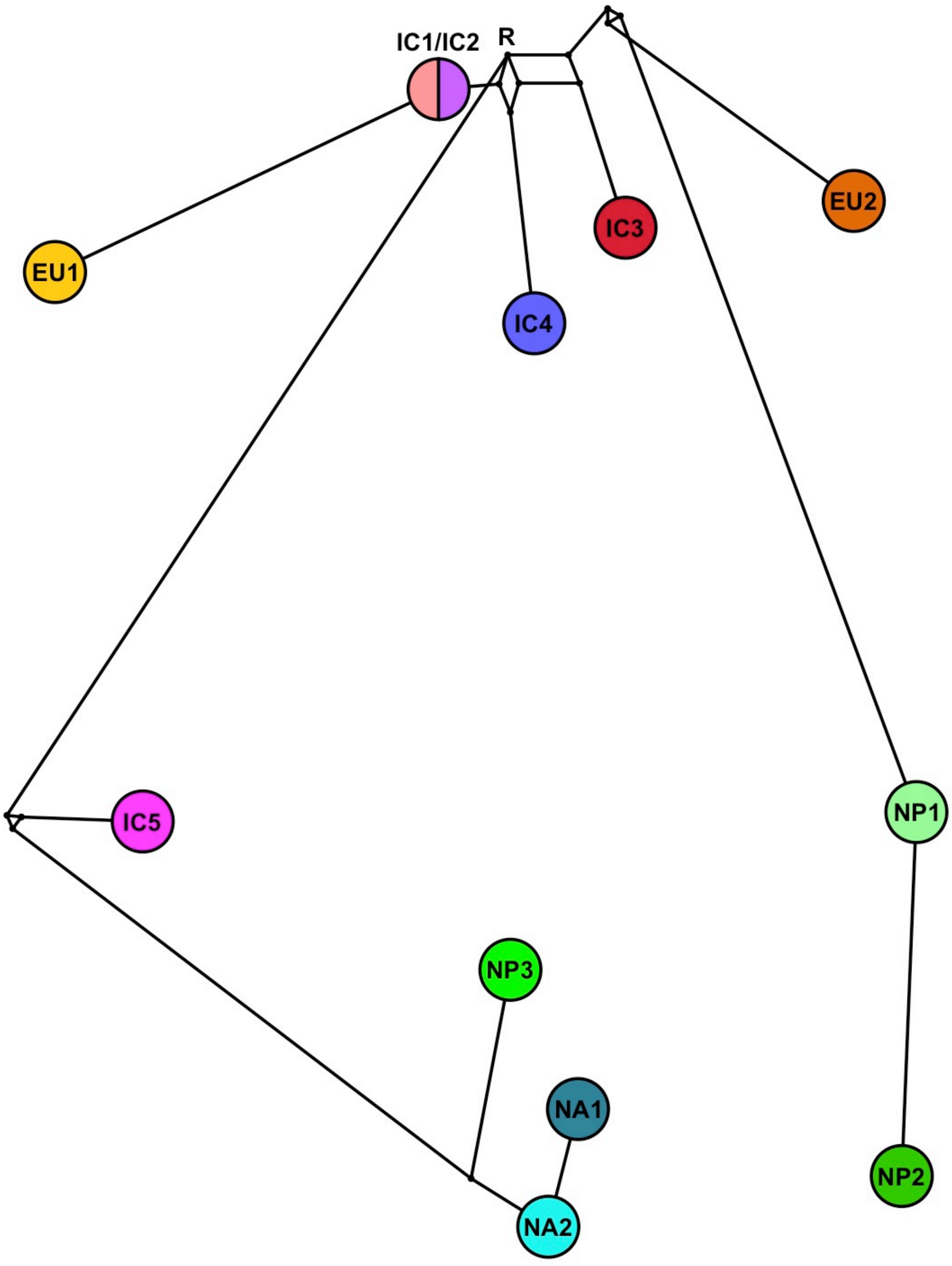

Supplementary Figure S5. Median-joining network (MJN) with tolerance ε = 10 for the five Indochinese lineages (IC1-IC5), the three Japanese lineages (NP1-NP3) and four known introduced lineages (EU1, EU2, NA1, NA2) of *Phytophthora ramorum* based on a nuclear seven–loci (*Avh120*, *Avh121*, *btub*, gwEuk.30.30.1, *hsp90*, ITS, *trp1*) dataset. The node with the highest number of connections and the most central position in this network and in the MJN with ε = 0 (Figure 9) was designated as root (R). Compared to the nuclear MJN with ε = 0 this MJN shows several alternative evolutionary pathways. Missing intermediates represent extant unsampled genotypes, extinct ancestors or "false-positives". Branch lengths are proportional to the number of mutations.

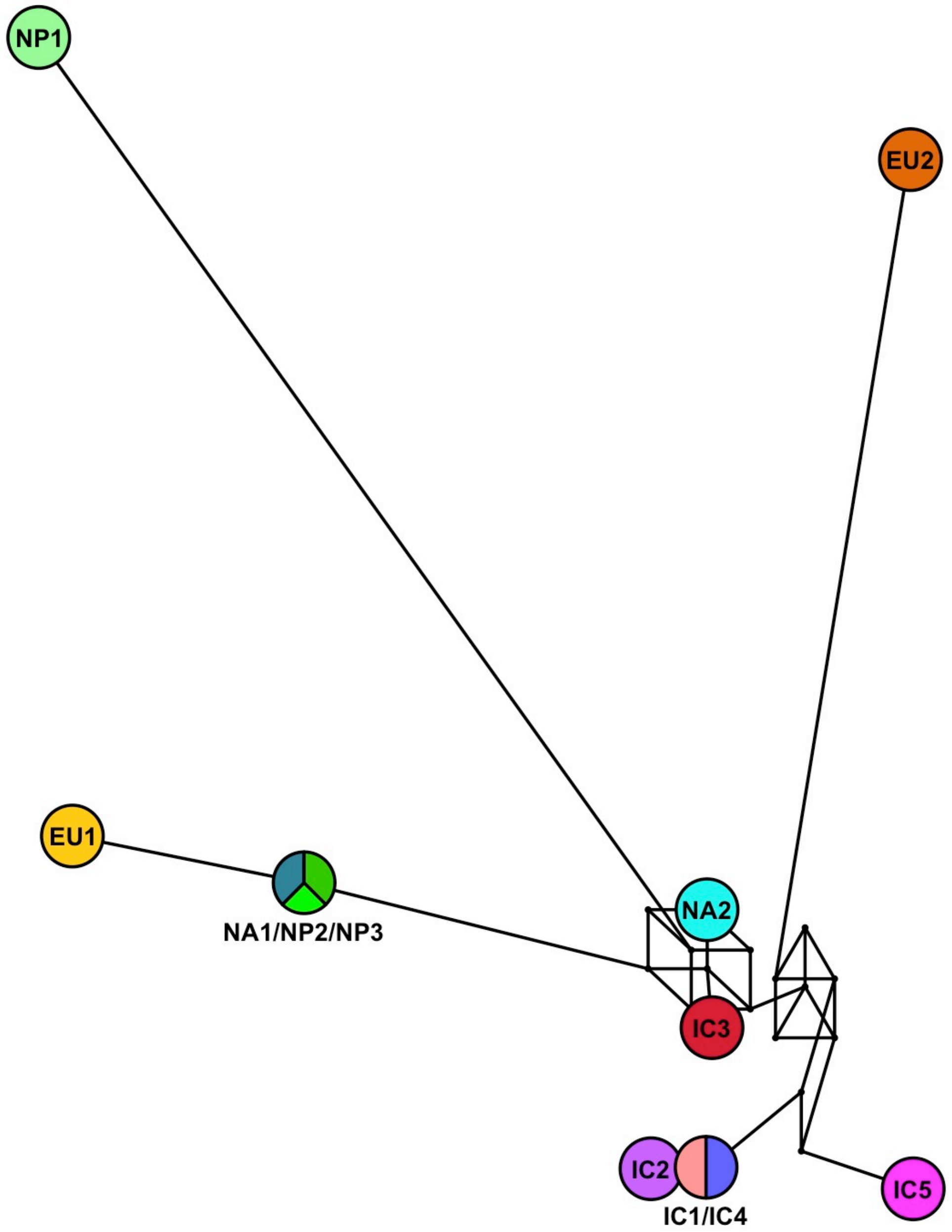

Supplementary Figure S6. Median-joining network (MJN) with tolerance ε = 10 for the five Indochinese lineages (IC1-IC5), the three Japanese linrages (NP1-NP3) and the four known lineages (EU1, EU2, NA1, NA2) of *Phytophthora ramorum* based on a mitochondrial five–loci (*cox1*, *cox2*, *nadh1*, Prv8, Prv9) dataset. Compared to the mitochondrial MJN with ε = 0 this MJN contains multiple alternative evolutionary pathways and is too complex for selecting a root. Missing intermediates represent extant unsampled genotypes, extinct ancestors or "false-positives". Branch lengths are proportional to the number of mutations.
