# Supplementary material for: The Destructive Tree Pathogen Phytophthora ramorum Originates from the Laurosilva Forests of East Asia
Source: J Fungi (Basel). 2021 Mar 18;7(3):226. doi: 10.3390/jof7030226 (PMC8003361; doi:10.3390/jof7030226)
Supplement: Supplementary file 1 [file jof-07-00226-s001.zip › Jung et al. 2021_Supplementary Tables S1-S4.pdf]

Supplementary Tables

Supplementary Table S1. Location, altitude and vegetation of the forest stream catchments in northern Vietnam and Japan included in the *Phytophthora ramorum* survey, sampling method, lineages, mating types (A1, A2, A0) and isolate codes.

Stream catchment no.	GPS coordinates	Altitude (m a.s.l)	Location; stream/river	Vegetation of catchment	Sampling method	*Phytophthora ramorum* lineages/mating types: isolate codes [a]
VN-S1	N22 20.127<br>E103 46.782	2083	Northern Vietnam, Hoàng Liên NP, Fansipan mountain; forest stream	Subalpine and montane Rhododendron scrub and forests, montane diverse laurosilva forests dominated by *Castanopsis* spp., *Quercus* spp. and *Neolitsea* spp.	Stream baiting	**IC1/A1:** VN71, VN75, VN78, VN118, VN120, VN121, VN122, VN123, VN280, VN281, VN283, VN284
					Fallen leaves/ flowers from stream	**IC1/A1:** VN835, VN876, VN900, VN901<br>**IC1/n.t.:** VN833, VN836, VN837, VN856, VN857, VN864, VN880, VN882, VN904, VN914<br>**IC5/A0**: VN863
VN-S2	N22 20.440<br>E103 46.576	2007	Northern Vietnam, Hoàng Liên NP, Fansipan mountain; forest stream	Subalpine and montane Rhododendron scrub and forests, montane diverse laurosilva forests dominated by *Castanopsis* spp., *Quercus* spp. and *Neolitsea* spp.	Stream baiting	**IC1/A1**: VN160
					Fallen leaves/ flowers from stream	**IC1/A1:** VN866, VN877, VN878, VN879, VN881, VN889, VN921, VN924<br>**IC1/n.t.:** VN865, VN909, VN912, VN915, VN918
VN-S3	N22 21.046<br>E103 46.273	1913	Northern Vietnam, Hoàng Liên NP, Fansipan mountain; forest stream	Montane diverse laurosilva forests dominated by *Castanopsis* spp., *Quercus* spp. and *Neolitsea* spp.	Stream baiting	**IC2/A2:** VN142, VN150, VN169, VN314
VN-S4	N22 21.029<br>E103 46.317	1904	Northern Vietnam, Hoàng Liên NP, Fansipan mountain; forest stream	Montane diverse laurosilva forests dominated by *Castanopsis* spp., *Quercus* spp. and *Neolitsea* spp.	Stream baiting	**IC1/A1**: VN57<br>**IC3/A2:** VN88
VN-S5	N22 20.906<br>E103 46.197	1895	Northern Vietnam, Hoàng Liên NP, Fansipan mountain; forest stream	Montane diverse laurosilva forests dominated by *Castanopsis* spp., *Quercus* spp. and *Neolitsea* spp.	Stream baiting	**IC1/A1**: VN163, VN166, VN201
VN-S6	N 22 20.911,<br>E 103 46.199	1896	Northern Vietnam, Hoàng Liên NP, Fansipan mountain; forest stream	Montane diverse laurosilva forests dominated by *Castanopsis* spp., *Quercus* spp. and *Neolitsea* spp.	Stream baiting	-

Stream catchment no.	GPS coordinates	Altitude (m a.s.l)	Location; stream/river	Vegetation of catchment	Sampling method	*Phytophthora ramorum* lineages/mating types: isolate codes [a]
VN-S7	N22 20.902 E103 46.261	1912	Northern Vietnam, Hoàng Liên NP, Fansipan mountain; forest stream	Montane diverse laurosilva forests dominated by *Castanopsis* spp., *Quercus* spp. and *Neolitsea* spp.	Stream baiting<br>Fallen Rhododendron leaves on forest ground	**IC1/A1**: VN216, VN217, VN310, VN313, VN407, VN408, VN409<br>**IC1/A1**: VN831, VN832
VN-S8	N 22 20.904, E 103 46.259	1911	Northern Vietnam, Hoàng Liên NP, Fansipan mountain; forest stream	Montane diverse laurosilva forests dominated by *Castanopsis* spp., *Quercus* spp. and *Neolitsea* spp.	Stream baiting	-
VN-S9	N22 18.597 E103 52.426	1013	Northern Vietnam, close to Hoàng Liên NP; Muong Hoa River	Subalpine and montane Rhododendron scrub and forests, montane diverse laurosilva forests, rice fields	Stream baiting	-
VN-S10	N 22 19.372, E 103 49.780	1193	Northern Vietnam, Hoàng Liên NP, Fansipan mountain; Cat Cat River	Montane diverse laurosilva forests dominated by *Castanopsis* spp., *Quercus* spp. and *Neolitsea* spp.	Stream baiting<br>Fallen leaves from stream	-<br>**IC1/A1**: VN852, VN853, VN1015, VN1016
VN-S11	N 22 22.230, E 103 52.615	1308	Northern Vietnam, Sau Chua mountain; forest stream, tributary of Ngòi Duôi River	Montane diverse laurosilva forest with *Chamaecyparis hodginsii*, *Cunninghamia lanceolata* plantations	Stream baiting<br>Fallen leaves from stream	-<br>**IC4/A1:** VN851
JP-S9	N 32 44.453, E 132 59.967	324	Japan, Shikoku, Tosashimizu area, Sada-yama forest; forest stream	Laurosilva forest dominated by *Castanopsis sieboldii*, *Quercus acuta* and *Machilus thunbergii*	Stream baiting	-
JP-S10	N 32 44.434, E 132 59.947	319	Japan, Shikoku, Tosashimizu area, Sada-yama forest; forest stream	Laurosilva forest dominated by *C. sieboldii*, *Q. acuta* and *M. thunbergii*	Stream baiting	-
JP-S11	N 32 44.354, E 132 59.962	288	Japan, Shikoku, Tosashimizu area, Sada-yama forest; forest stream	Laurosilva forest dominated by *C. sieboldii*, *Q. acuta* and *M. thunbergii*	Stream baiting	-
JP-S12	N 32 46.333, E 132 58.174	44	Japan, Shikoku, Tosashimizu area, Sada-yama forest; forest stream	Laurosilva forest dominated by *C. sieboldii*, *Q. acuta* and *M. thunbergii*	Stream baiting	-
JP-S13	N 33 8.757, E 132 55.400	401	Japan, Shikoku, Shimanto area; forest stream	Laurosilva forests dominated by *Quercus salicina*, *Q. sessilifolia*, *Abies firma*, *Chamaecyparis obtusa* and *Tsuga sieboldii*; *Cryptomeria japonica* plantations	Stream baiting	-

Stream catchment no.	GPS coordinates	Altitude (m a.s.l)	Location; stream/river	Vegetation of catchment	Sampling method	*Phytophthora ramorum* lineages/mating types: isolate codes [a]
JP-S14	N 33 8.708, E 132 55.102	537	Japan, Shikoku, Shimanto area; forest stream	Laurosilva forest dominated by *Q. salicina*, *Q. sessilifolia*, *A. firma*, *C. obtusa* and *T. sieboldii*	Stream baiting	-
JP-S15	N 33 8.810, E 132 55.022	523	Japan, Shikoku, Shimanto area; forest stream	Laurosilva forests dominated by by *Q. salicina*, *Q. sessilifolia*, *A. firma*, *C. obtusa* and *T. sieboldii*	Stream baiting<br>Fallen leaves from stream	-<br>**NP1/A1:** JP236, JP716, JP916; *PL*, *PFL*
JP-S16	N 33 9.644, E 132 56.186	264	Japan, Shikoku, Shimanto area; Okutome River	Laurosilva forests dominated by by *Q. salicina*, *Q. sessilifolia*, *A. firma*, *C. obtusa* and *T. sieboldii*; *Cryptomeria japonica* plantations	Stream baiting	-
JP-S17	N 32 1.961, E 131 12.569	190	Japan, Kyushu, Aya area; forest stream, tributary of Ayakita River	Laurosilva forest dominated by *Castanopsis cuspidata*, *Quercus glauca* and *M. thunbergii*	Stream baiting	-
JP-S18	N 32 1.607, E 131 13.199	51	Japan, Kyushu, Aya area; Ayakita River	Laurosilva forests dominated by *C. cuspidata*, *Q. glauca* and *M. thunbergii*; *Cryptomeria japonica* plantations	Stream baiting	-
JP-S19	N 32 22.339, E 131 8.686	609	Japan, Kyushu, Shiba area; forest stream, tributary of Hitotsuse River	Partially deciduous montane forest dominated by *Quercus* spp., *Carpinus japonica* and *A. firma*; *Cr. japonica* plantations	Stream baiting	-
JP-S20	N 32 21.502, E 131 10.252	1014	Japan, Kyushu, Shiba area; Shitayabu River	Partially deciduous montane forest dominated by *Quercus* spp., *Carpinus japonica*, *Fagus crenata*, *Betula grossa*, *Aesculus turbida*, *Pieris japonica* and *A. firma*	Stream baiting	-*PFF*
JP-S21	N 32 22.045, E 131 8.774	612	Japan, Kyushu, Shiba area; forest stream, tributary of Hitotsuse River	Partially deciduous montane forest dominated by *Quercus* spp., *Ca. japonica*, *F. crenata*, *B. grossa*, *A. turbida*, *P. japonica* and *A. firma*	Stream baiting	-
JP-S22	N 31 30.966, E 130 47.004	546	Japan, Kyushu, Tarumizu area; forest stream	Laurosilva forest dominated by *Lithocarpus edulis* and *Castanopsis sieboldii*	Stream baiting<br>Fallen leaves from stream	**NP2/A2:** JP387, JP462

Stream catchment no.	GPS coordinates	Altitude (m a.s.l)	Location; stream/river	Vegetation of catchment	Sampling method	*Phytophthora ramorum* lineages/mating types: isolate codes [a]
JP-S23	N 31 31.975, E 130 45.829	505	Japan, Kyushu, Tarumizu area; Kushira River	Laurosilva forest dominated by *C. sieboldii*, *Zelkova serrata*, *M. thunbergii* and *C. obtusa*	Stream baiting	-
JP-S24	N 31 31.547, E 130 49.537	203	Japan, Kyushu, Tarumizu area; forest stream, tributary of Kushira River	Laurosilva forests dominated by *C. sieboldii*, *Zelkova serrata*, *M. thunbergii* and *C. obtusa*; *Cr. japonica* plantations	Stream baiting	-
JP-S31	N 31 49.732, E 130 38.468	335	Japan, Kyushu, Kirishima area; forest stream	Laurosilva forest dominated by *Quercus* spp. and *Neolitsea sericea*; also *Camelia japonica*	Fallen leaves from stream<br>Fallen leaves from forest floor	**NP1/A1:** JP1202<br>**NP3/A2:** JP975; *PL*

[a] n.t., not tested; *PL*, *Phytophthora lateralis* isolated from necrotic *Chamaecyparis* foliage collected from the forest floor; *PFL*, *Phytophthora foliorum* isolated from fallen leaf collected from the stream; *PFF, P. foliorum* isolated from attached necrotic foliage of *Pieris japonica*. For a complete list of all *Phytophthora* and other oomycete species isolated in the Vietnamese survey see Jung *et al.* (2020). The surveys in Kyushu and Shikoku were part of a larger oomycete survey across Japan and a complete list of all *Phytophthora* and other oomycete species isolated will be presented elsewhere. Details of the 12 isolates from the EU1, EU2, NA1 and NA2 lineage isolates used in this study are given in Franceschini *et al.* (2014).

Supplementary Table S2. GenBank accession numbers of seven nuclear and five mitochondrial gene regions generated in this study for 35 isolates from 12 lineages of *Phytophthora ramorum* and one isolate of *P. lateralis* and whole genome shotgun sequence contig numbers of GenBank assembly accession GCA_012656075.1 for *P. hibernalis* isolate BL41G.

**Species**	**Isolate**	**Lineage**	***GenBank accession numbers**											
			**ITS**	***btub***	***hsp90***	***Avh120***	***Avh121***	**gwEuk. 30.30.1**	***trp1***	***cox1***	**cox2**	***nadh1***	**Prv8**	**Prv9**
P. ramorum	VN57	IC1	***MN861673***	MW323680	MW323716	MW323752	MW323788	MW323616	MW323580	***MN861129***	MW323823	MW323859	MW323894	MW323931
P. ramorum	VN160	IC1	MW300302	MW323684	MW323720	MW323756	MW323792	MW323620	MW323584	MW323654	MW323827	MW323863	MW323896	MW323935
P. ramorum	VN283	IC1	MW300303	MW323686	MW323722	MW323758	MW323794	MW323622	MW323586	MW323655	MW323829	MW323865	MW323898	MW323937
P. ramorum	VN313	IC1	MW300304	MW323687	MW323723	MW323759	MW323795	MW323623	MW323587	MW323656	MW323830	MW323866	MW323899	MW323938
P. ramorum	VN831	IC1	MW300306	MW323689	MW323725	MW323761	MW323797	MW323625	MW323589	MW323658	MW323832	MW323868	MW323901	MW323940
P. ramorum	VN833	IC1	MW300307	MW323690	MW323726	MW323762	MW323798	MW323626	MW323590	MW323659	MW323833	MW323869	MW323902	MW323941
P. ramorum	VN852	IC1	***MN861672***	MW323692	MW323728	MW323764	MW323800	MW323628	MW323592	***MN861128***	MW323835	MW323871	MW323903	MW323943
P. ramorum	VN866	IC1	MW300310	MW323694	MW323730	MW323766	MW323802	MW323630	MW323594	MW323662	MW323837	MW323873	MW323904	MW323945
P. ramorum	VN1015	IC1	MW300311	MW323695	MW323731	MW323767	MW323803	MW323631	MW323595	MW323663	MW323838	MW323874	MW323905	MW323946
P. ramorum	VN142	IC2	MW300300	MW323682	MW323718	MW323754	MW323790	MW323618	MW323582	MW323652	MW323825	MW323861	MW323919	MW323933
P. ramorum	VN150	IC2	MW300301	MW323683	MW323719	MW323755	MW323791	MW323619	MW323583	MW323653	MW323826	MW323862	MW323920	MW323934
P. ramorum	VN169	IC2	***MN861674***	MW323685	MW323721	MW323757	MW323793	MW323621	MW323585	***MN861130***	MW323828	MW323864	MW323897	MW323936
P. ramorum	VN314	IC2	MW300305	MW323688	MW323724	MW323760	MW323796	MW323624	MW323588	MW323657	MW323831	MW323867	MW323900	MW323939
P. ramorum	VN88	IC3	***MN861675***	MW323681	MW323717	MW323753	MW323789	MW323617	MW323581	***MN861131***	MW323824	MW323860	MW323895	MW323932
P. ramorum	VN851	IC4	MW300308	MW323691	MW323727	MW323763	MW323799	MW323627	MW323591	MW323660	MW323834	MW323870	MW323921	MW323942
P. ramorum	VN863	IC5	MW300309	MW323693	MW323729	MW323765	MW323801	MW323629	MW323593	MW323661	MW323836	MW323872	MW323922	MW323944
P. ramorum	JP236	NP1	MW300293	MW323673	MW323709	MW323745	MW323781	MW323609	MW323573	MW323645	MW323816	MW323852	MW323888	MW323924
P. ramorum	JP716	NP1	MW300296	MW323676	MW323712	MW323748	MW323784	MW323612	MW323576	MW323648	MW323819	MW323855	MW323891	MW323927
P. ramorum	JP916	NP1	MW300297	MW323677	MW323713	MW323749	MW323785	MW323613	MW323577	MW323649	MW323820	MW323856	MW323892	MW323928
P. ramorum	JP1202	NP1	MW300299	MW323679	MW323715	MW323751	MW323787	MW323615	MW323579	MW323651	MW323822	MW323858	MW323918	MW323930
P. ramorum	JP387	NP2	MW300294	MW323674	MW323710	MW323746	MW323782	MW323610	MW323574	MW323646	MW323817	MW323853	MW323889	MW323925
P. ramorum	JP462	NP2	MW300295	MW323675	MW323711	MW323747	MW323783	MW323611	MW323575	MW323647	MW323818	MW323854	MW323890	MW323926
P. ramorum	JP975	NP3	MW300298	MW323678	MW323714	MW323750	MW323786	MW323614	MW323578	MW323650	MW323821	MW323857	MW323893	MW323929
P. ramorum	P1367	EU1	MW300312	MW323696	MW323732	MW323768	MW323804	MW323632	MW323596	MW323664	MW323839	MW323875	MW323906	MW323947
P. ramorum	P1376	EU1	MW300313	MW323697	MW323733	MW323769	MW323805	MW323633	MW323597	MW323665	MW323840	MW323876	MW323907	MW323948
P. ramorum	P1578	EU1	***MN861668***	MW323698	MW323734	MW323770		MW323634	MW323598	***MN861124***	MW323841	MW323877	MW323908	MW323949
P. ramorum	P2460	EU2	***MN861669***	MW323699	MW323735	MW323771	MW323806	MW323635	MW323599	***MN861125***	MW323842	MW323878	MW323909	MW323950
P. ramorum	P2461	EU2	MW300314	MW323700	MW323736	MW323772	MW323807	MW323636	MW323600	MW323666	MW323843	MW323879	MW323910	MW323951
P. ramorum	P2462	EU2	MW300315	MW323701	MW323737	MW323773	MW323808	MW323637	MW323601	MW323667	MW323844	MW323880	MW323911	MW323952
P. ramorum	P1403	NA1	MW300316	MW323702	MW323738	MW323774	MW323809	MW323638	MW323602	MW323668	MW323845	MW323881	MW323912	MW323953
P. ramorum	P1421	NA1	***MN861670***	MW323703	MW323739	MW323775	MW323810	MW323639	MW323603	***MN861126***	MW323846	MW323882	MW323913	MW323954
P. ramorum	P1496	NA1	MW300317	MW323704	MW323740	MW323776	MW323811	MW323640	MW323604	MW323669	MW323847	MW323883	MW323914	MW323955
P. ramorum	P2056	NA2	***MN861671***	MW323705	MW323741	MW323777	MW323812	MW323641	MW323605	***MN861127***	MW323848	MW323884	MW323915	MW323956
P. ramorum	P2057	NA2	MW300318	MW323706	MW323742	MW323778	MW323813	MW323642	MW323606	MW323670	MW323849	MW323885	MW323916	MW323957
P. ramorum	P2058	NA2	MW300319	MW323707	MW323743	MW323779	MW323814	MW323643	MW323607	MW323671	MW323851	MW323886	MW323917	MW323958
P. hibernalis	BL41G		contig 172	contig 227	contig 121	contig 345	contig 49	contig 10	contig 33	contig 44	contig 44	contig 139	contig 139	contig 139

Species	Isolate	Lineage	*GenBank accession numbers											
			**ITS**	***btub***	***hsp90***	***Avh120***	***Avh121***	**gwEuk. 30.30.1**	***trp1***	***cox1***	**cox2**	***nadh1***	**Prv8**	**Prv9**
P. lateralis	P2451	UK	MW300320	MW323708	MW323744	MW323780	MW323815	MW323644	MW323608	MW323672	MW323850	MW323887	MW323923	MW323959

Isolate details for the IC1-IC5 and NP1-NP3 lineages are given in Supplementary Table S1. Origin of known lineage isolates: EU1 isolates, *Viburnum* and *Rhododendron* in the UK; EU2 isolates, *Larix* spp. in Northern Ireland, UK; NA1 isolates, *Rhododendron* and *Vaccinium* in California and Oregon, USA; NA2 isolates, unknown host plants in Oregon, Washington and California, USA (for details see Franceschini *et al.* (2014). For details of *P. lateralis* isolate P2451 see Brasier et al. (2012). With isolate P1578 the amplification of *Avh121* failed repeatedly.

Supplementary Table S3. Details of primers, annealing temperatures and Mg^{2+} concentrations used to amplify the genomic regions investigated in this study.

Locus	Primer names	Primer sequences (5′-3′)	Primer (μM)	Annealing temperature (°C)	Mg^{2+} (mM)	Reference for primer sequences
Nuclear gene regions						
rDNA ITS [a]	ITS1<br>ITS4	TCCGTAGGTGAACCTGCGG<br>TCCTCCGCTTATTGATATGC	0.5	57	2	White et al. (1990)
Avh120 [a]	PrRXLR120_-242F<br>PrRXLR120_639R	CTGTGCGAATCTTGCAACC<br>GCGAATCATTCCTACGCTTG	0.2	55	2	Goss et al. (2009)
Avh121 [a]	PrRXLR121_-244F<br>PrRXLR121_623R	CATGCTGACCCATTCAGTACC<br>TCAAAGGGCCTTCTGCATAC	0.2	55	2	Goss et al. (2009)
btub [b]	TUBUF2<br>TUBUR1	CGGTAACAACTGGGCCAAGG<br>CCTGGTACTGCTGGTACTCAG	0.8	60	1.5	Kroon et al. (2004)
gwEuk.30.30.1 [a]	Pr9C3c_F<br>Pr9C3c_R	TTCAAGTGGGAGGAGAGGAA<br>GCTCCAGGATGATCTCTCCA	0.2	55	2	Goss et al. (2009)
hsp90 [a]	HSP90F1<br>HSP90R1	GCTGGACACGGACAAGAACC<br>ACACCCTTGACRAACGACAG	1	68	2	Blair et al. (2008)
trp1 [a]	Trp1F1<br>Trp1R1	CCGAACACGCAAAGTGCAAATAACAAGATGGG<br>CGTCACGGCTGAGCGAAAGTCCCCG	0.2	60	2	Goss et al. (2009)
Mitochondrial gene regions						
cox1 [b]	COXF4N<br>COXR4N	GTATTTCTTCTTTATTAGGTGC<br>CGTGAACTAATGTTACATATAC	0.8	60	3.5	Kroon et al. (2004)
	FM80RC [c]<br>FM85	TTTCAACAAATCATAAAGATATT<br>AACTTGACTAATAATACCAAA	0.5	55	2	Martin & Tooley (2003)
cox2 + spacer [a]	FM35<br>Phy10b	CAGAACCTTGGCAATTAGG<br>GCAAAAGCACTAAAAATTAAATATAA	0.4	55	2	Martin (2008)
nadh1 [b]	NADHF1<br>NADHR1	CTGTGGCTTATTTTACTTTAG<br>CAGCAGTATACAAAAACCAAC	0.8	60	3.5	Kroon et al. (2004)
Prv8 [b]	Prv8-F<br>Prv8-R	GTTCTCATTAATTCTTGTTGA<br>TTTATATTTGGTGTAGTGATG	0.2	52	4	Martin (2008)
Prv9 [a]	Prv9-F<br>Prv9-R	CTATTAACATTTAAGATAGTCCA<br>GAACAATTTGAAATTTTACCTA	0.2	55	2	Martin (2008)

[a] Loci amplified using DreamTaq DNA Polymerase (EP0701, Thermo Fisher Scientific Inc.) supplied with 10X DreamTaq buffer containing 20 mM MgCl2.

[b] Loci amplified using *Taq* DNA Polymerase, recombinant (EP0401, Thermo Fisher Scientific Inc.) supplied with 10X *Taq* Buffer containing KCl. [c] Primer FM80RC is the reverse complement of FM80. Primers were synthetized by Bio Basic Canada Inc. (Markham, ON) and Elizabeth Pharmacon spol. s.r.o. (Brno, Czech Republic).

Supplementary Table S4. Summary of main traits discriminating the 12 *Phytophthora ramorum* lineages from Indochina (IC), Japan (NP), Europe (EU) and North America (NA).

**Lineage (mating type)**	**IC1** (A1)	**IC2** (A2)	**IC3** (A2)	**IC4** (A1)	**IC5** (A0)	**NP1** (A1)	**NP2** (A2)	**NP3** (A2)	**EU1** (A1)	**EU2** (A1)	**NA1** (A2)	**NA2** (A2)
**IC1** (A1)		C-28-M-(NU)[a]-(MI)	C-M-NU-MI	C-20-NU	C-28-M-NU-MI	C-NU-MI	C-20-28-M-NU-MI	C-28-M-NU-MI	C-28-NU-MI	(C)-NU-MI	C-20-28-M-NU-MI	C-(28)-M-NU-MI
**IC2** (A2)	C-28-M-*(NU)-(MI)		C-28-NU-MI	C-20-28-M-NU-(MI)	C-M-NU-MI	C-28-M-NU-MI	C-NU-MI	C-28-NU-MI	C-(28)-M-NU-MI	C-28-M-NU-MI	C-20-NU-MI	C-28-NU-MI
**IC3** (A2)	C-M-NU-MI	C-28-NU-MI		C-28-M-NU-MI	C-28-M-NU-MI	C-M-NU-MI	C-28-NU-MI	C-28-NU-MI	C-28-M-NU-MI	C-28-M-NU-MI	C-28-NU-MI	C-28-NU-MI
**IC4** (A1)	C-20-NU	C-20-28-M-NU-(MI)	C-28-M-NU-MI		C-20-28-M-NU-MI	C-20-28-NU-MI	C-28-M-NU-MI	C-20-M-NU-MI	C-20-28-NU-MI	C-NU-MI	C-20-28-M-NU-MI	C-20-M-NU-MI
**IC5** (A0)	C-28-M-NU-MI	C-M-NU-MI	C-28-M-NU-MI	C-20-28-M-NU-MI		C-28-M-NU-MI	C-M-NU-MI	C-28-M-NU-MI	C-(28)-M-NU-MI	C-28-M-NU-MI	C-M-NU-MI	C-28-M-NU-
**NP1** (A1)	C-NU-MI	C-28-M-NU-MI	C-M-NU-MI	C-20-28-NU-MI	C-28-M-NU-MI		C-20-28-M-*(NU)-MI	C-28-M-NU-MI	C-28-NU-MI	C-20-28-NU-MI	C-28-M-NU-MI	C-28-M-NU-MI
**NP2** (A2)	C-20-28-M-NU-MI	C-NU-MI	C-28-NU-MI	C-20-28-M-NU-MI	C-M-NU-MI	C-20-28-M-*(NU)-MI		C-28-NU-	C-20-(28)-M-NU-MI	C-28-M-NU-MI	C-20-(28)-NU-	C-20-28-NU-MI
**NP3** (A2)	C-28-M-NU-MI	C-28-NU-MI	C-28-NU-MI	C-20-M-NU-MI	C-28-M-NU-MI	C-28-M-NU-MI	C-28-NU-		C-28-M-NU-MI	C-M-NU-MI	C-28-NU-	(C)-NU-MI
**EU1** (A1)	C-28-NU-MI	C-(28)-M-NU-MI	C-28-M-NU-MI	C-20-28-NU-MI	C-(28)-M-NU-MI	C-28-NU-MI	C-20-(28)-M-NU-MI	C-28-M-NU-MI		C-20-28-NU-MI	C-(28)-M-NU-MI	C-28-M-NU-MI
**EU2** (A1)	(C)-20-NU-MI	C-28-M-NU-MI	C-28-M-NU-MI	C-NU-MI	C-28-M-NU-MI	C-20-28-NU-MI	C-28-M-NU-MI	C-M-NU-MI	C-20-28-NU-MI		C-20-28-M-NU-MI	(C)-M-NU-MI
**NA1** (A2)	C-20-28-M-NU-MI	C-20-(28)-NU-MI	C-28-NU-MI	C-20-28-M-NU-MI	C-(28)-M-NU-MI	C-28-M-NU-MI	C-20-(28)-NU-	C-28-NU-	C-(28)-M-NU-MI	C-20-28-M-NU-MI		C-28-NU-MI
**NA2** (A2)	C-(28)-M-NU-MI	C-28-NU-MI	C-28-NU-MI	C-20-M-NU-MI	C-28-M-NU-MI	C-28-M-NU-MI	C-20-28-NU-MI	(C)-NU-MI	C-28-M-NU-MI	(C)-M-NU-MI	C-28-NU-MI	

C, colony morphology on carrot agar (CA) at 20°C; 20, growth rate on CA at 20°C; 28, G × E stress test on 2% V8-juice agar at 28°C; M, mating type; NU, concatenated nuclear gene sequences; MI, concatenated mitochondrial sequences.

[a] Only heterozygous positions different.
